# Adiponectin receptor 1-mediated stimulation of Cav3.2 channels in trigeminal ganglion neurons induces nociceptive behaviors in mice

**DOI:** 10.1186/s10194-023-01658-2

**Published:** 2023-08-25

**Authors:** Yuan Zhang, Yuan Wei, Tingting Zheng, Yu Tao, Yufang Sun, Dongsheng Jiang, Jin Tao

**Affiliations:** 1 Clinical Research Center of Neurological Disease & Department of Geriatrics, The Second Affiliated Hospital of Soochow University, 1055 San-Xiang Road, Suzhou, 215004 People’s Republic of China; 2 Jiangsu Key Laboratory of Neuropsychiatric Diseases, Soochow University, Suzhou, 215123 People’s Republic of China; 3 Department of Physiology and Neurobiology & Centre for Ion Channelopathy, Suzhou Medical College of Soochow University, 199 Ren-Ai Road, Suzhou, 215123 People’s Republic of China; 4 Institute of Regenerative Biology and Medicine, Helmholtz Zentrum München, 81377 Munich, Germany

**Keywords:** Adiponectin receptor 1, Trigeminal ganglion neurons, Cav3.2 channels, Pain

## Abstract

**Background:**

Adipokines, including adiponectin, are implicated in nociceptive pain; however, the underlying cellular and molecular mechanisms remain unknown.

**Methods:**

Using electrophysiological recording, immunostaining, molecular biological approaches and animal behaviour tests, we elucidated a pivotal role of adiponectin in regulating membrane excitability and pain sensitivity by manipulating Cav3.2 channels in trigeminal ganglion (TG) neurons.

**Results:**

Adiponectin enhanced T-type Ca^2+^ channel currents (*I*_T_) in TG neurons through the activation of adiponectin receptor 1 (adipoR1) but independently of heterotrimeric G protein-mediated signaling. Coimmunoprecipitation revealed a physical association between AdipoR1 and casein kinase II alpha-subunits (CK2α) in the TG, and inhibiting CK2 activity by chemical inhibitor or siRNA targeting CK2α prevented the adiponectin-induced *I*_T_ response. Adiponectin significantly activated protein kinase C (PKC), and this effect was abrogated by CK2α knockdown. Adiponectin increased the membrane abundance of PKC beta1 (PKCβ1). Blocking PKCβ1 pharmacologically or genetically abrogated the adiponectin-induced *I*_T_ increase. In heterologous expression systems, activation of adipoR1 induced a selective enhancement of Cav3.2 channel currents, dependent on PKCβ1 signaling. Functionally, adiponectin increased TG neuronal excitability and induced mechanical pain hypersensitivity, both attenuated by T-type channel blockade. In a trigeminal neuralgia model induced by chronic constriction injury of infraorbital nerve, blockade of adipoR1 signaling suppressed mechanical allodynia, which was prevented by silencing Cav3.2.

**Conclusion:**

Our study elucidates a novel signaling cascade wherein adiponectin stimulates TG Cav3.2 channels via adipoR1 coupled to a novel CK2α-dependent PKCβ1. This process induces neuronal hyperexcitability and pain hypersensitivity. Insight into adipoR-Cav3.2 signaling in sensory neurons provides attractive targets for pain treatment.

**Supplementary Information:**

The online version contains supplementary material available at 10.1186/s10194-023-01658-2.

## Introduction

Adiponectin, originally termed adipocyte complement-associated protein of 30 kDa, is a cytokine secreted exclusively by differentiated adipocytes that plays crucial roles in modulating numerous physiological processes [1, 2]. By virtue of binding to its membrane receptors, including adiponectin receptor 1 (adipoR1) and adipoR2 [3], adiponectin exerts diverse biological effects, such as cardioprotection, antioxidative stress, and regulation of glucose and lipid metabolism [4–6]. Recently, emerging evidence has also revealed a pivotal role of adiponectin/adipoRs in nociceptive behaviors [7, 8]. Studies have shown that the *Adipor1* gene is potentially associated with the severity of postoperative pain but not cancer pain [9]. It has also been demonstrated that intrathecal administration of adiponectin might regulate carrageenan-induced inflammatory pain [10]. Moreover, adiponectin might regulate electroacupuncture-mediated analgesic effects through spinal adipoR-AMPK signaling in chronic inflammatory pain induced by complete Freund’s adjuvant (CFA) [11]. Interestingly, further findings come from clinical evidence that patients with headache or with pain in lower limb osteoarthritis have increased serum levels of adiponectin [12–14]. Nevertheless, direct proof and the detailed molecular mechanism underlying peripheral nociception of adiponectin remain elusive.

T-type Ca^2+^ channels (T-type channels) are low-voltage activated channels that are unique in their capacity to modulate neuronal excitability with minimal depolarization and play controlling functions in low-threshold exocytosis [15, 16]. Molecular cloning revealed the existence of three distinct α1 subunits of T-type channels, known as Cav3.1, Cav3.2 and Cav3.3, with unique pharmacological profiles and specific expression patterns in the brain and peripheral nervous system [17, 18]. Aberrant expression and/or function of α1 subunits of T-type channels are associated with pathological conditions such as the absence of epilepsy, seizure susceptibility, and pain perception [17, 19, 20]. In the peripheral pain pathways, T-type channels play pivotal roles in nociceptive signaling, as they not only change the action potential firing rate of nociceptive neurons and shape their firing patterns but also regulate quantal neurotransmitter release at dorsal horn synapses [21, 22]. According to growing evidence from genetic [23] and pharmacological analyses [16, 24–26], targeting T-type channels, in particular Cav3.2, has enormous therapeutic promise for the treatment of pain.

In the present study, we determined the role of adiponectin in regulating TG T-type channels and elucidated the underlying molecular mechanisms by which adiponectin functions as a nociceptive effector. Our findings demonstrate that adiponectin stimulates Cav3.2 channels through adipoR1 coupled to a novel CK2α-dependent PKCβ1 signaling cascade. This adipoR1-mediated signaling contributes to TG neuronal hyperexcitability and nociceptive behaviors in mice. Targeting adipoR1-mediated signaling might offer novel therapeutic strategies/targets for pain management.

## Materials and methods

### Dissociation of TG neurons

All experimental and surgical protocols were approved by the Institutional Animal Care and Use Committee of Soochow University and followed the National Institutes for Health guidelines for laboratory animal use. Trigeminal ganglion (TG) neurons were dissociated from ICR mice (8–10 weeks old, regardless of sex) as previously described [26–28]. Briefly, TGs were dissected out bilaterally and collected in Hank's balanced salt solution (HBSS). After removing connective tissue and trimming, the ganglia were incubated in a dissecting solution containing 2.25 mg/ml collagenase D (Merck) for 25 min and 1.25 mg/ml trypsin (Merck) for 20 min. Individual cells were dissociated by triturating the tissue through a fire-polished glass pipette and plated on Matrigel-coated glass cover slips. TG neurons were recorded 3–6 h after plating. We sorted TG neurons into small- (soma diameter < 25 μm) and medium-sized (soma diameter 25 to 35 μm) groups and made electrophysiological recordings in cells less than 25 μm because the majority of these are nociceptors [24, 29].

### Electrophysiology

Whole-cell patch clamp recordings were conducted at room temperature (22 ± 1 °C) as previously described [27]. The electrodes when filled with internal solution had a resistance between 3 and 5 MΩ. Signals were sampled at 50 kHz and low-pass filtered at 1 kHz (Model Digidata 1322A). The series resistance was compensated (75%), and membrane currents were recorded using pClamp 10.2 software (Molecular Devices). The intracellular solution for recording *I*_T_ contained (in mM): 110 CsCl, 0.3 Na_2_-GTP, 4 MgATP, 10 EGTA, and 25 HEPES (pH 7.4, osmolality 295 mOsm). The external solution for recording *I*_T_ contained (in mM): 140 TEA-Cl, 5 BaCl_2_, 5 CsCl, 0.5 MgCl_2_, 5.5 glucose, and 10 HEPES (pH 7.35, osmolality 305 mOsm). To separate *I*_T_, whole-cell recordings were conducted in external solution containing the L-type channel blocker nifedipine (5 μM), the N- and P/Q-type channel blocker ω-conotoxin MVIIC (0.2 μM), and the R-type channel blocker SNX-482 (0.2 μM). Currents were recorded at -40 mV by 40 ms depolarizing pulses from a holding potential of -110 mV. The two kinetically distinct Kv currents, *I*_A_ and *I*_DR,_ were separated following our previous protocols [30, 31]. Typical Kv currents exhibited transient components followed by slowly decaying and sustained components. A 150-ms prepulse to -10 mV was included to inactivate the transient channels, resulting in sustained *I*_DR_ isolation. Offline subtraction of *I*_DR_ from the total current yielded* I*_A_. For current clamp experiments and *I*_Na_ recordings, TG neurons were superfused with an external solution that contained (in mM) 2 KCl, 2 MgCl_2_, 128 NaCl, 2 CaCl_2_, 30 glucose and 25 HEPES (pH 7.4, osmolality 305 mOsm). The intracellular solution contained (in mM) 10 NaCl, 2 EGTA, 110 KCl, 0.3 Na_2_-GTP, 4 Mg-ATP, and 25 HEPES (pH 7.25, osmolality 300 mOsm). Adiponectin was applied to a patched neuron by an air-pressure microinjector (PV830, Pneumatic Picopump, World Precision Instruments) through a glass pipette, the tip of which was placed 15–25 μm from the soma of TG neurons. In cells dialyzed with compounds, the resistances of electrode pipettes were 2 to 3 MΩ for intracellular delivery. In siRNA-mediated knockdown experiments, small TG neurons with green fluorescence were chosen for electrophysiological recordings.

### Immunoblotting

Immunoblotting was conducted as described previously [27, 32]. The membranes were probed with primary antibodies against adipoR1 (rabbit, 1:1000, Abcam), adipoR2 (rabbit, 1:1000, Abcam), RACK1 (rabbit, 1:1000, Abcam), PKCK2α (rabbit, 1:1000, Cell Signaling Technology), CaMKII (rabbit, 1:1000, Abcam), phospho-CaMKII (mouse, 1:800, Abcam), PKCβ1 (mouse, 1:1000, ThermoFisher Scientific), Cav3.2 (rabbit, 1:800, Alomone) and GAPDH (rabbit, 1:3000, Cell Signaling Technology). Blots were washed and subsequently probed with horseradish peroxidase (HRP)-conjugated goat anti-rabbit or goat anti-mouse IgG secondary antibody (1:5000, Abcam). The immunocomplexes were detected with enhanced chemiluminescence (Merck Millipore). The Chin-X Imager System (Shanghai, China) was used to detect the bands, and NIH ImageJ software was used to quantify the protein band intensities.

### Coimmunoprecipitation

Coimmunoprecipitation analysis was conducted as previously described [32]. In brief, total proteins were extracted from TG tissues with Pierce IP lysis buffer supplemented with a protease and phosphatase inhibitor cocktail (Thermo Scientific). Extracts containing 400–500 μg of protein were incubated with 3 μg of antibody against adipoR1 (rabbit, 1:500, Abcam), followed by incubation with protein A–Sepharose beads (Amersham Biosciences). Immunoprecipitates were washed three times, and the bound proteins were eluted by boiling in loading buffer and subjected to sodium dodecyl sulfate–polyacrylamide gel electrophoresis (SDS‒PAGE). Proteins were analyzed by immunoblotting using the indicated primary antibodies.

### Immunohistochemistry

Immunostaining analysis was conducted as previously described [27, 32, 33]. Briefly, TGs were sectioned (15-μm thickness) on a cryostat (Leica CM1950), permeabilized with Triton X-100, and blocked with 5% goat serum. After washing, TG sections were incubated with primary antibodies against adipoR1 (rabbit, 1:300, Abcam), NeuN (mouse, 1:500, Cell Signaling Technology), GS (mouse, 1:500, Abcam), NF200 (mouse, 1:500, Sigma‒Aldrich), and CGRP (mouse, 1:300, Abcam) and visualized using IB_4_-fluorescein isothiocyanate (FITC) (Sigma‒Aldrich) or the appropriate secondary antibodies, including FITC-conjugated donkey anti-mouse IgG (1:300, ThermoFisher Scientific) and Cy3-conjugated donkey anti-rabbit IgG (1:300, Merck Millipore). Fluorescence images were captured utilizing a Nikon104c microscope equipped with a CoolSnap-ProColor CCD camera (Photometrics).

### Measurement of PKC activity

PKC activity was assayed using the PKC Kinase Activity Assay kit (Abcam) as described previously [26, 32]. Briefly, TG cells in the plates were pretreated with either CK2α-siRNA or its negative control NC-siRNA and then stimulated by 100 nM adiponectin for 30 min. Subsequently, lysis buffer was added, and the lysed TG cells were collected, sonicated and centrifuged. The supernatant was used for PKC activity assays, which were performed according to the supplier’s instructions.

### Cell culture and transient transfection

The human adipoR1 cDNA obtained from Origene was cloned into the pCMV6-AC-GFP vector. The full-length α_1_-subunits of Cav3.1, Cav3.2, and Cav3.3 were cloned into the pcDNA3. The human cDNA clones of α_1_G, α_1_H, and α_1_I were gifted from Prof. Terrance P. Snutch from the University of British Columbia. As previously described [28, 34, 35], human embryonic kidney 293 (HEK293) cells were cultured following standard protocols and transfected using Lipofectamine 3000 (Invitrogen). Cells were used for patch clamp recording two days after transfection.

### Reverse transcription-PCR (RT-PCR)

After extraction of the total RNA using Takara RNAiso Plus, the quality and RNA concentrations of the samples were assessed using a NanoDrop spectrophotometer (NanoDrop One, Thermo Fisher Scientific). Purified RNAs were reverse-transcribed by the PrimeScript RT Reagent Kit (Takara) according to the manufacturer’s protocol. PCR was performed in a 25 μl reaction mixture as described previously [27, 32, 36]. The primers used in this study are summarized in Tables S1 and S2.

### Analysis of PKCβ1 translocation

Immunofluorescence analysis of translocation was conducted as described in our previous studies [26]. In brief, TG neurons were treated with 100 nM adiponectin for 30 min and then fixed with 4% PFA. After incubation with PBS containing 10% goat serum and 0.2% Triton X-100, neurons were probed with mouse anti-PKCβ1 (1:500, Abcam) overnight at 4 °C and visualized with FITC-conjugated donkey anti-mouse IgG (1:300, ThermoFisher Scientific). The images were captured with a Zeiss LSM510 confocal microscope and analyzed with Image-Pro Plus v6.0 analysis software (Media Cybernetics).

### Animal model and behavioral studies

Animals were housed in a temperature-controlled environment under a 12/12-h light–dark cycle with ad libitum access to food and water and were habituated to laboratory conditions for 3 days prior to the experiments. All efforts were made to minimize animal suffering and the number of animals used. A trigeminal neuralgia model was established by chronic constriction injury of the infraorbital nerve (CCI-ION) in mice as previously described [27, 37]. In brief, the mice were anesthetized with isoflurane, and the left infraorbital nerve was separated near the infraorbital foramen. Fine forceps and a bent tip needle loaded with a silk suture (4–0 silk) were used to place two loose ligatures around the ION 1–2 mm apart. Sham-operated mice were subjected to a similar surgical procedure without ligation. The investigators were blinded to the treatment assignment during behavioral tests. As described previously [28, 33], the escape threshold of mechanical sensitivity was determined with *von* Frey filaments with bending forces ranging from 0.008 g to 2 g (Ugo Basile, Italy). Stimuli were gently applied to the skin within the infraorbital nerve territory, near the center of the vibrissal pad. A 22-gauge needle was introduced through the foramen rotundum, infraorbital canal, and infraorbital foramen to administer intra-TG injections. The needle’s tip terminated at the medial portion of the TG, and 3 μl of reagents were slowly injected over a five-minute period. Small interfering RNA (siRNA) for adipoR1 (adipoR1-siRNA), RACK1-siRNA, CK2α-siRNA, Cav3.2-siRNA, and scrambled negative controls (Ribo Biological Technology), tagged with 6-carboxyfluorescein (6-FAM), were chemically modified by 5’-cholesteryl and 2’-O-methyl, and were administrated daily for 2 consecutive days. Three days following the siRNA intra-TG injection, immunoblotting was performed to assess the efficacy of the siRNA knockdown.

### Pharmacological reagents

All pharmacological agents were obtained from Sigma–Aldrich unless otherwise indicated. Stock solutions of adiponectin, the PKCβ1 inhibitory peptide (PKCβ1-IP, Santa Cruz Biotechnology), the PKCβ2 inhibitory peptide (PKCβ2-IP, Santa Cruz Biotechnology) and GDP-β-S were prepared with double deionized water (Merck Milli-Q). Stock solutions of CX-4945 (Selleck), TBB (Abcam), nitrendipine, KT-5720 (Calbiochem), GF109203X, Bisindolylmaleimide V, Gö6976 (Tocris Bioscience), LY333531 (Tocris Bioscience), HBDDE, TTA-P2 (Alomone Labs), and Z941 (from T.P. Snutch) were prepared in dimethyl sulfoxide (DMSO). The DMSO concentration in each medium was less than 0.05% and did not have significant effects on *I*_T_.

### Data analysis and statistics

Data are presented as the mean ± S.E.M. Microsoft Excel, Prism 8.0, and Clampfit 10.2 were used for data collection and statistical analysis. Paired or unpaired *t* tests were performed to compare two groups as appropriate. One-way analysis of variance (ANOVA) was applied to compare the statistics of different groups, and if not otherwise mentioned, subsequent Bonferroni correction was applied. Two-way repeated-measures ANOVA with a post hoc Bonferroni test was used to evaluate the behavioral data. A *p* value less than 0.05 was considered statistically significant. Sigmoidal dose–response curves of adiponectin were fitted to the data using the following Hill equation: I/I_control_ = 1/[1 + 10^(logEC50−X)^*n*], where EC_50_ is the concentration at which the half-maximum effect occurs, X values are logarithms of concentrations, and *n* is the coefficient. The Boltzmann equation was used to fit plots showing voltage-dependent activation and steady-state inactivation.

## Results

### Adiponectin enhances T-type channel currents in TG neurons

**Fig. 1:**
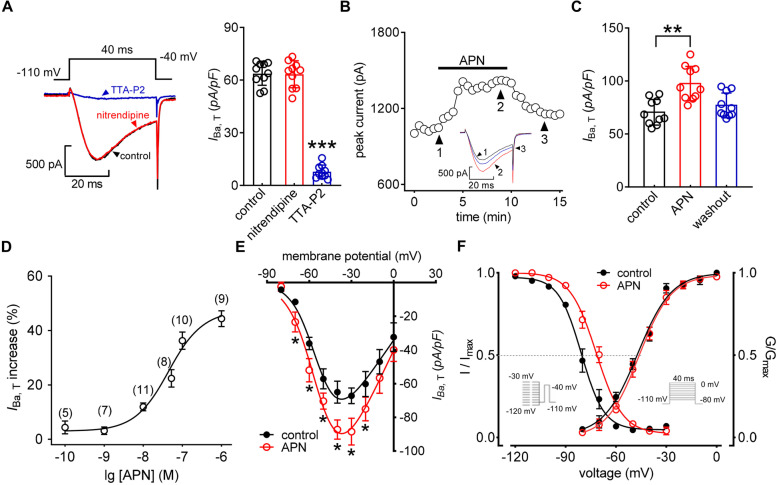
Adiponectin enhances *I*_T_ in small-sized TG neurons. **A** Representative traces (*left*) and bar chart (*right*) indicating the effect of nitrendipine (5 μM,* n* = 10 cells) or TTA-P2 (3 µM,* n* = 10 cells) on *I*_T_. *Inset*s indicate the stimulation waveform. ***p < 0.001 (*vs.* pre-TTA-P2), paired *t* test. **B** Time course of *I*_T_ changes induced by 100 nM adiponectin (APN). *Inset*s indicate representative current traces. Numbers represent points used for exemplary traces. **C** Bar chart showing the enhancement of *I*_T_ induced by 100 nM adiponectin (*n* = 10 cells). **p < 0.01 (*vs.* control), paired *t* test. **D** Dose‒response curves of adiponectin on *I*_T_. The solid line represents the best fit with the *Hill* equation. In brackets is the number of cells recorded at each concentration of adiponectin. **E** Current–voltage curve indicating the enhancement of 100 nM adiponectin on *I*_T_ current density (*n* = 11 cells). *I*_T_ was recorded from the holding potential of -110 mV with depolarizing pulses ranging from -80 to + 40 mV in 10-mV increments. *p < 0.05 (*vs.* control), one-way ANOVA. **F** Adiponectin at 100 nM did not alter the voltage-dependent activation properties of *I*_T_ (*n* = 11 cells) but shifted steady-state inactivation properties in a depolarizing direction (*n* = 12 cells). *Inset*s indicate stimulation protocols

Whole-cell recordings in this study were restricted to small-diameter (< 25 μm) TG neurons because they play pivotal roles in nociceptive processing [24, 27, 29]. Currents were elicited by 40 ms depolarizing pulses from -110 mV to -40 mV. As described in our previous studies [26, 27, 38], a cocktail of channel blockers, including nifedipine (5 μM, L-type channel blocker), SNX482 (0.2 μM, R-type channel blocker), and ω-conotoxin MVIIC (0.2 μM, N- and P/Q-type channel blocker), was bath-applied before recording to separate T-type channel currents (hereafter, *I*_T_). The isolated inward currents were not further affected by nitrendipine (5 μM, 1.8 ± 1.1%), a well-described L-type channel blocker, but were dramatically suppressed by TTA-P2 (3 µM, 92.5 ± 3.6%), a specific T-type channel blocker [39], demonstrating the effective *I*_T_ isolation (Fig. 1A). When 100 nM adiponectin was applied to TG neurons, the peak amplitude of *I*_T_ was significantly increased and was partially reversed within 5 min after adiponectin was removed by washout (Figs. 1B and C). We further investigated the dose‒response relationship of adiponectin on *I*_T_ and observed an EC_50_ of 47.1 nM by fitting the data to a sigmoidal *Hill* equation (Fig. 1D). Moreover, the biophysical characteristics of T-type channels affected by adiponectin were also examined. Adiponectin at 100 nM enhanced the peak inward current density from -65.1 ± 8.7 pA/pF to -87.3 ± 5.2 pA/pF at -40 mV (Fig. 1E) and altered the voltage dependence of channel inactivation. A depolarizing shift of ~ 8.3 mV in the half-maximal inactivation potential was observed (*V*_half_ from -78.5 ± 7.1 mV to -70.2 ± 6.6 mV, Fig. 1F), while the *V*_half_ of the voltage dependence of activation remained unchanged (*V*_half_ from -48.5 ± 7.1 mV to -51.3 ± 8.3 mV, Fig. 1F).

### AdipoR1 mediates the adiponectin-induced *IT* response

**Fig. 2:**
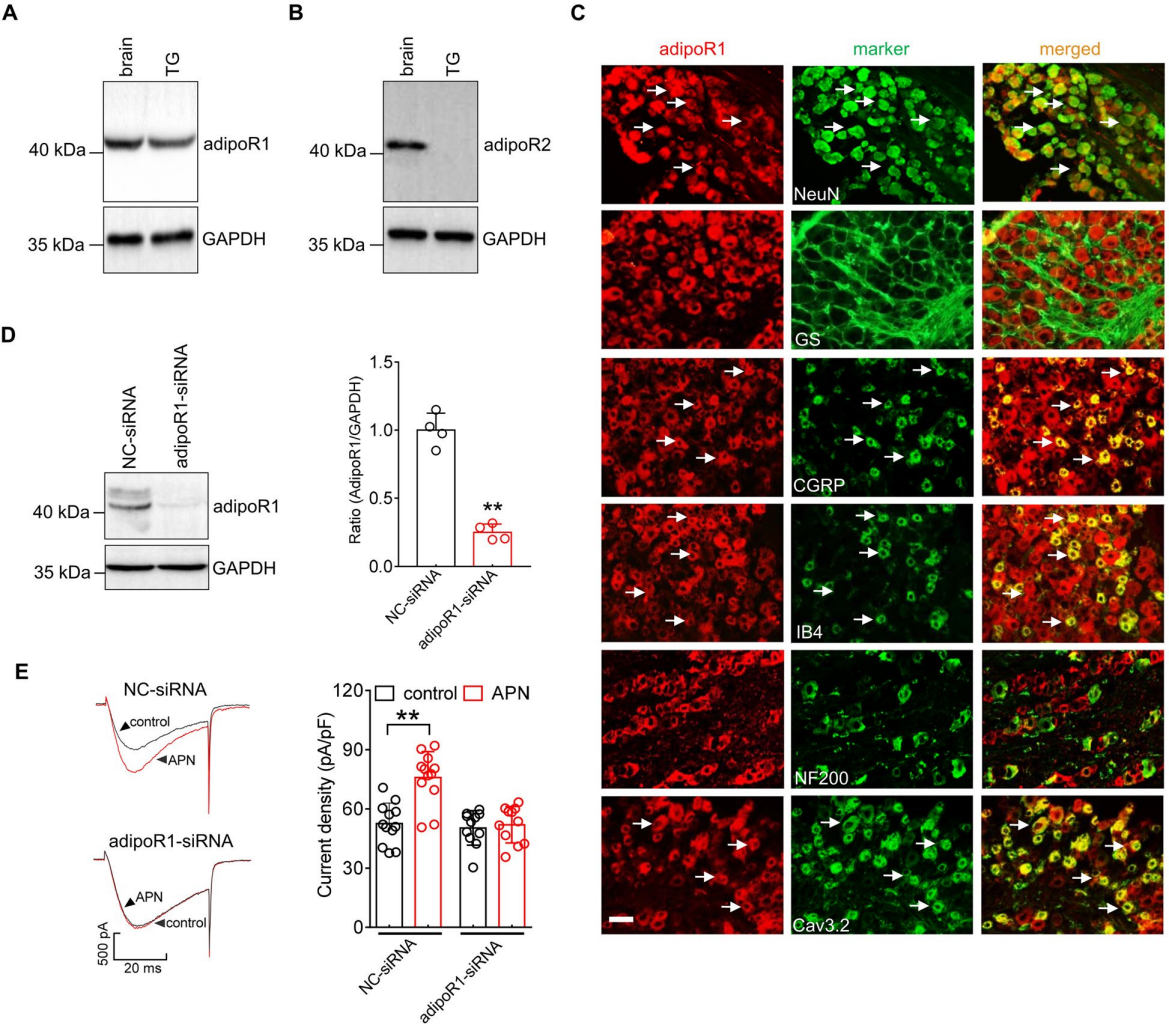
AdipoR1 mediates the adiponectin-induced increase in *I*_T_. **A**, **B** Protein abundance of adipoR1 (A) and adipoR2 (B) in the brain and TGs of intact mice. GAPDH was used as an equal loading control. Representative blots of at least 3 independent experiments are shown. **C**, Colocalization of adipoR1 (*red*) with NeuN, GS, NF200, CGRP, IB_4_ or Cav3.2 (*green*) in TG sections. Arrows show the colocalization. Scale bar, 50 µm. **D**, Protein abundance of adipoR1 in TG cells treated with control siRNA (NC-siRNA) or adipoR1 siRNA (adipoR1-siRNA). Representative blots of at least 3 independent experiments are shown. **p < 0.01 (*vs.* NC-siRNA), unpaired *t* test. **E**, Representative traces (*left*) and bar chart (*right*) revealing that adipoR1-siRNA treatment prevented the adiponectin-induced *I*_T_ increase (*n* = 11 cells). Adiponectin at 100 nM significantly enhanced *I*_T_ in cells transduced with NC-siRNA (*n* = 12 cells). **p < 0.01 (*vs.* control + NC-siRNA), unpaired *t* test

The adiponectin receptor 1 (adipoR1) and adipoR2 subtypes have been identified as functional receptors for adiponectin in mammals [40]. Thus, we examined the involvement of precise adipoR in the adiponectin-induced *I*_T_ response. Immunoblot analysis demonstrated that adipoR1 (Fig. 2A & Fig. S1) was endogenously expressed in the TG, whereas adipoR2 was not detected (Fig. 2B & Fig. S1). Further immunostaining of TG tissue sections showed that adipoR1 was coexpressed with the neuronal marker NeuN but was not expressed in glutamine synthetase-labeled satellite glial cells (Fig. 2C). We subsequently differentiated small unmyelinated peptidergic or nonpeptidergic neurons and medium and large myelinated neurons by a variety of phenotypic markers, including calcitonin gene-related peptide (CGRP), isolectin B4 (IB4) and 200 kDa neurofilament protein (NF200). Immunostaining analysis revealed that adipoR1 was coexpressed with IB4 and CGRP but exhibited comparatively little colocalization with NF200 (Fig. 2C). The Cav3.2 isoform is the predominant T-type channel expressed in primary sensory neurons and is associated with nociceptive processing [32, 41, 42]. Further double staining analysis indicated that adipoR1 was heavily colocalized with Cav3.2 in TG neurons (Fig. 2C). We next examined the participation of adipoR1 in the adiponectin-mediated *I*_T_ increase. Since there is a lack of commercially available specific inhibitors for adipoR1, a 2’-O-methyl-modified and 5’-cholesteryl-modified siRNA-mediated knockdown approach was applied to determine the effect of adiponectin on* I*_T_ in adipoR1-siRNA-transduced TG neurons. Compared to administration of control siRNA (NC-siRNA), intra-TG injection of chemically modified adipoR1-siRNA significantly decreased the protein abundance of adipoR1 (Fig. 2D & Fig. S2). Knockdown of adipoR1 abrogated the adiponectin–induced *I*_T_ response (increase 3.8 ± 1.5%, Fig. 2E), while adiponectin at 100 nM still robustly increased *I*_T_ in NC-siRNA-treated groups (increase 32.7 ± 6.1%, Fig. 2E).

### The AdipoR1-induced *IT* increase is dependent on the protein kinase CK2α

**Fig. 3:**
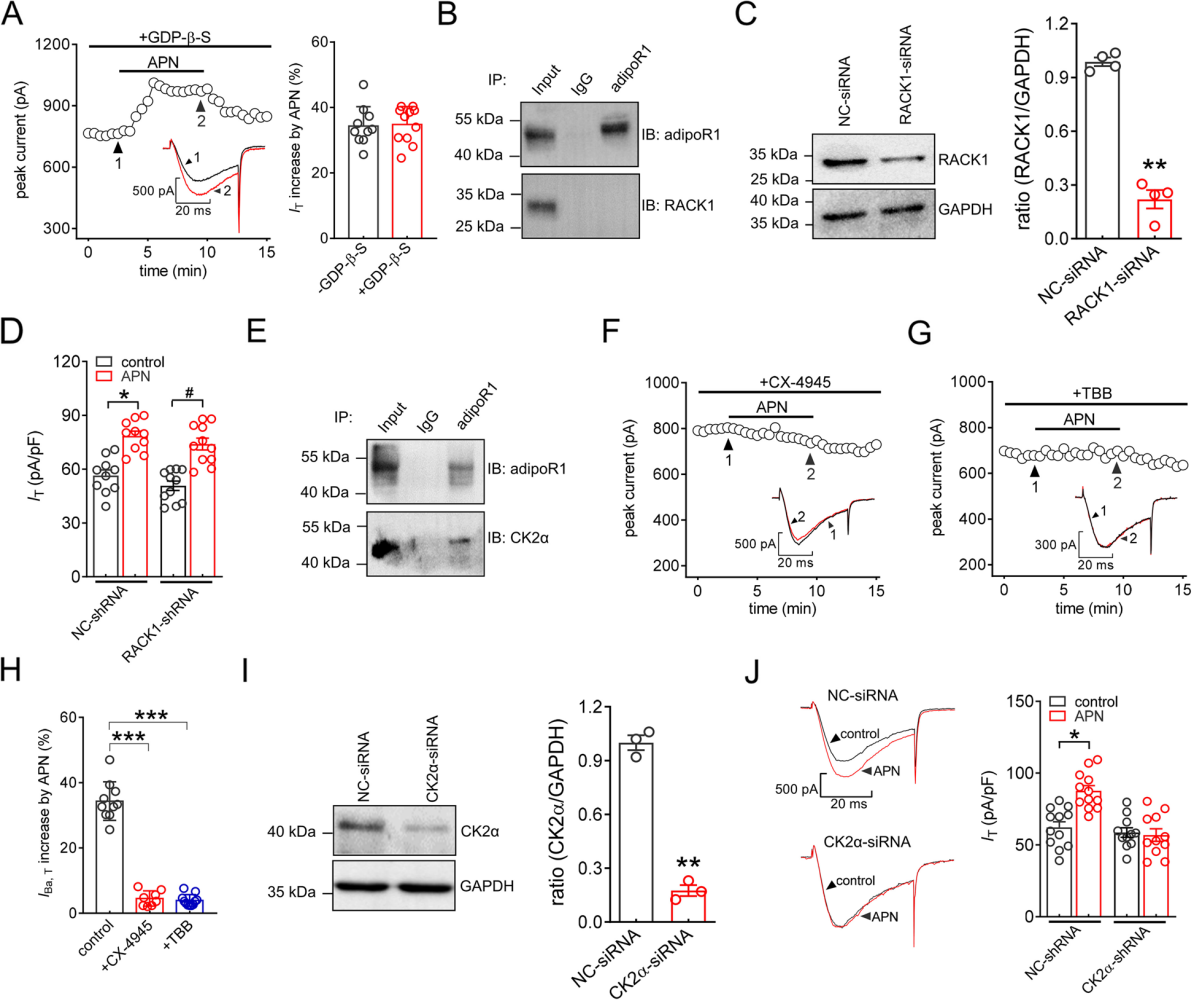
The AdipoR1-induced *I*_T_ increase is mediated by the protein kinase CK2α. **A** Time course of *I*_T_ changes (*left*) and bar chart (*right*) showing the effect of adiponectin at 100 nM on *I*_T_ in cells dialyzed with GDP-β-S (1 mM) (*n* = 11 cells). *Inset*s indicate representative current traces. Numbers represent points used for exemplary traces. **B** Coimmunoprecipitation of adipoR1 with RACK1 in the TG. Representative blots of at least 3 independent experiments are shown. **C** Protein abundance of RACK1 in TG cells treated with RACK1-siRNA or NC-siRNA. Representative blots of at least 3 independent experiments are shown. **p < 0.01 (*vs.* NC-siRNA), unpaired *t* test. **D** Summary of results revealing that treatment with either NC-siRNA (*n* = 10 cells) or RACK1-siRNA (*n* = 11 cells) did not affect the adiponectin-induced *I*_T_ response. *p < 0.05 (*vs.* control + NC-siRNA), ^#^*p* < 0.05 (*vs.* control + RACK1-siRNA), unpaired *t* test. **E** Interaction of adipoR1 with PKCK2α in the TG. Representative blots of at least 3 independent experiments are shown. **F-G** Time course of *I*_T_ changes showing the effect of adiponectin at 100 nM on *I*_T_ in cells preincubated with CX-4945 (*F*, 10 μM) or TBB (*G*, 20 μM). *Inset*s indicate representative current traces. Numbers represent points used for exemplary traces. **H** Summary of the results revealing the effect of adiponectin at 100 nM on *I*_T_ in the presence of CX-4945 (*n* = 8 cells) or TBB (*n* = 11 cells). ***p < 0.001 (*vs.* control), paired *t* test. **I** Protein abundance of CK2α in NC-siRNA- or CK2α-siRNA-treated groups. Representative blots of at least 3 independent experiments are shown. **p < 0.01 (*vs.* NC-siRNA), unpaired *t* test. **J** Representative traces (*left*) and bar chart (*right*) revealing that treatment with CK2α-siRNA prevented the adiponectin-induced *I*_T_ increase (*n* = 11 cells). Adiponectin at 100 nM significantly enhanced *I*_T_ in cells transduced with NC-siRNA (*n* = 12 cells). *p < 0.05 (*vs.* control + NC-siRNA), unpaired *t* test

We next determined the underlying molecular mechanisms of the adipoR1-mediated *I*_T_ increase. The two adiponectin receptors belong to a novel group of membrane receptors that contain seven transmembrane domains, similar to those in G protein-coupled receptors, but are both structurally and functionally distinct [40]. Indeed, intracellular application of GDP-β-S (1 mM), a nonhydrolysable GDP analog that competitively inhibits G proteins, did not affect the adiponectin-induced *I*_T_ response (increase 34.7 ± 3.9%, Fig. 3A), excluding the possible participation of G proteins. Recent reports have suggested that by interacting with adipoR1 in HepG2 cells, receptor for activated C kinase 1 (RACK1), a cytosolic scaffold protein, may play a crucial bridging role in the transmission of adiponectin signaling [43]. Surprisingly, in the TG of mice, the association determined by coimmunoprecipitation showed that adipoR1 did not interact with RACK1 (Fig. 3B & Fig. S3), although RACK1 was endogenously expressed. In support of this, treatment of TG neurons with RACK1-specific siRNA (Fig. 3C & Fig. S4) did not affect the 100 nM adiponectin–induced *I*_T_ response (increase 30.9 ± 5.7%, Fig. 3D). Protein kinase casein kinase II (CK2) has been reported to participate in adiponectin receptor signaling [44]. We thus determined the role of CK2 in the adiponectin-induced *I*_T_ increase. Coimmunoprecipitation analysis of mouse TGs showed that CK2 α-subunits interacted with adipoR1 (Fig. 3E & Fig. S5), and pretreating TG neurons with the selective CK2 inhibitor CX-4945 abrogated the adiponectin-induced stimulatory effects in *I*_T_ (increase 4.5 ± 0.8%, Figs. 3F and H). This finding was further confirmed by pretreating TG neurons with 4,5,6,7-tetrabromobenzotriazole (TBB, increase 3.9 ± 0.5%, Fig. 3G and H), another CK2 inhibitor structurally unrelated to CX-4945. Further support for this assumption has been provided by investigation of adiponectin on *I*_T_ in CK2α-siRNA transduced neurons. Compared to NC-siRNA, the protein abundance of TG CK2α was dramatically decreased in CK2α-siRNA-treated groups (Fig. 3I & Fig. S6). Knockdown of CK2α completely abolished the adiponectin-induced increase in *I*_T_ (Fig. 3J).

### The adipoR1-induced *IT* response requires classical PKC isoforms

**Fig. 4:**
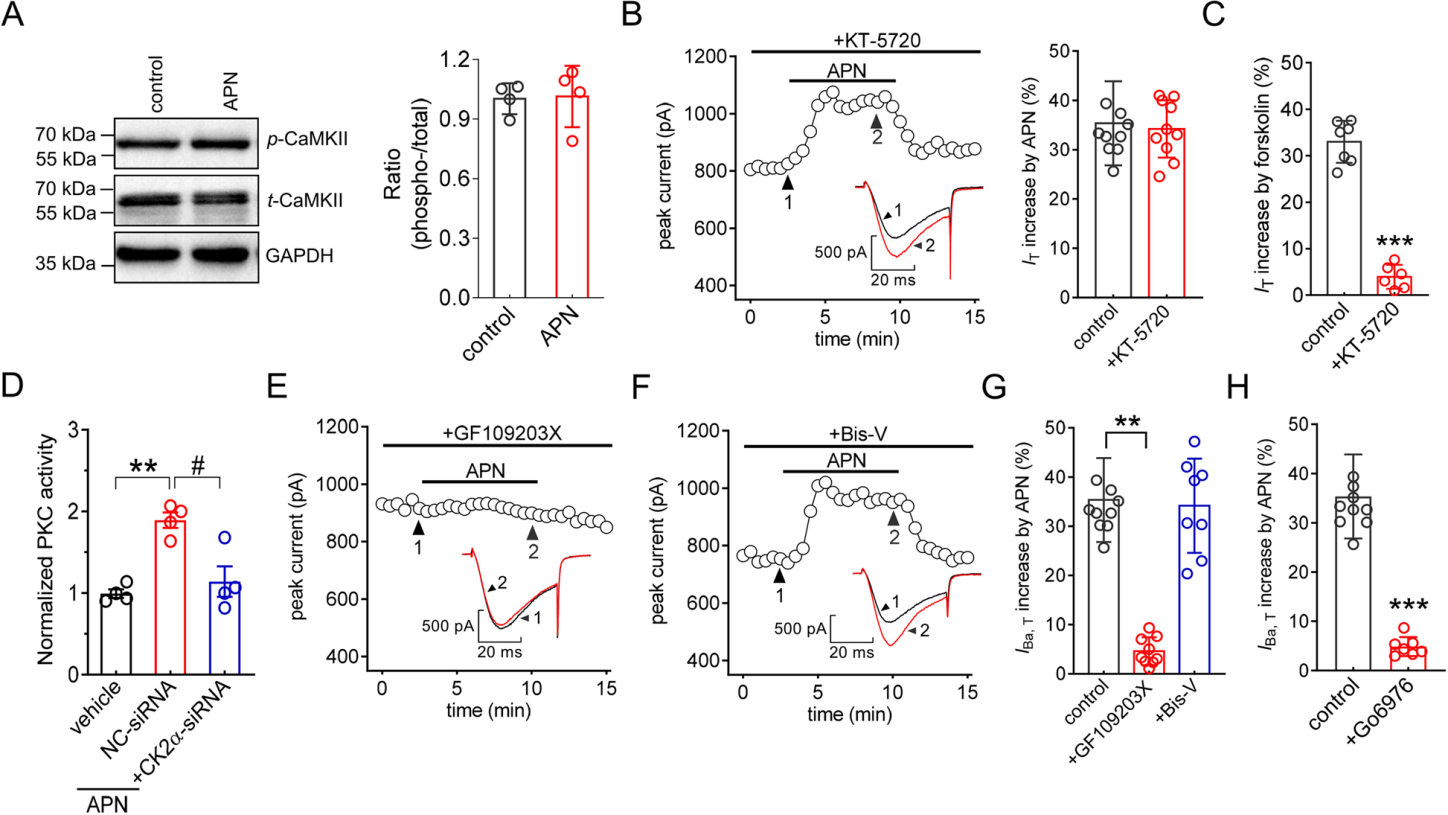
PKCβ1 is involved in the adiponectin-mediated *I*_T_ response. **A** Protein abundance of phosphorylated CaMKII (*p*-CaMKII) or total CaMKII (*t*-CaMKII) in TG cells treated with 100 nM adiponectin. Representative blots of at least 3 independent experiments are shown. **B,** Time course of *I*_T_ changes (*left*) and bar chart (*right*) revealing the effect of adiponectin at 100 nM on *I*_T_ in cells preincubated with 1 μM KT-5720 (*n* = 10 cells). *Inset*s indicate representative current traces. Numbers represent points used for exemplary traces. **C,** Bar graph revealing that pretreatment of cells with 1 μM KT-5720 abrogated the 20 µM forskolin-induced* I*_T_ response (*n* = 6 cells). ***p < 0.001 (*vs.* control), paired *t* test. **D,** Bar graph indicating that pretreatment of TG cells with CK2α-siRNA prevented the 100 nM adiponectin-induced increase in PKC activity. Data are means ± SEM from four independent experiments. **p < 0.01 (*vs.* vehicle), ^#^*p* < 0.05 (*vs.* NC-siRNA + APN), unpaired *t* test. **E–F,** Time course of *I*_T_ changes mediated by 100 nM adiponectin in cells pretreated with GF109203X (1 µM,* D*) or bisindolylmaleimide V (Bis-V, 1 µM,* E*). *Inset*s indicate representative current traces. Numbers represent points used for exemplary traces. **G,** Bar graph revealing the effect of 100 nM adiponectin on *I*_T_ in cells pretreated with GF109203X (*n* = 9 cells) or Bis-V (*n* = 8 cells). **p < 0.01 (*vs.* control), paired *t* test. **H** Bar graph revealing the effect of 100 nM adiponectin on *I*_T_ in the presence of Go6976 (200 nM, *n* = 7 cells). ***p < 0.001 (*vs.* control), paired *t* test

Evidence suggests that CaMKII signaling is a critical factor in adiponectin-induced biological activities [45]. However, treatment of TG cells with adiponectin at 100 nM did not change the protein abundance of phosphorylated CaMKII (*p-*CaMKII); the protein expression level of total CaMKII (*t-*CaMKII) also remained unchanged (Fig. 4A & Fig. S7). PKA was shown to function as a downstream target of CK2 [46] and is crucial for regulating the activity of T-type channels [47]. Thus, we examined the involvement of PKA in the adiponectin-induced *I*_T_ increase and found that pretreating neurons with the PKA-specific inhibitor KT-5720 (1 μM) did not affect the adiponectin-induced *I*_T_ response (increase 34.2 ± 5.8%, Fig. 4B). KT5720 (1 μM) utilized in the present study was effective in inhibiting PKA activity, as it abrogated the ability of forskolin (20 μM) to increase *I*_T_ (increase 3.6 ± 2.6%, Fig. 4C). Evidence has demonstrated the participation of a PKC–dependent regulatory contribution in T-type channels [47–49]. Analysis of PKC activity in TG cells revealed that 100 nM adiponectin robustly enhanced PKC activity; this effect was abrogated by CK2α-siRNA pretreatment (Fig. 4D). Moreover, pretreating TG neurons with bisindolylmaleimide I (GF109203X), a potent inhibitor of all PKC isoforms, completely prevented the adiponectin-induced *I*_T_ increase (Fig. 4E and G), while its inactive analog bisindolylmaleimide V elicited no such effects (Fig. 4F and G). Moreover, application of Go6976, a selective antagonist of the classical PKC isoforms, was observed to have a similar preventive effect on the adiponectin effect (Fig. 4H), which suggested that classic PKC isoforms might participate in adiponectin responses.

### PKCβ1 is involved in the adipoR1-induced *IT* response

**Fig. 5:**
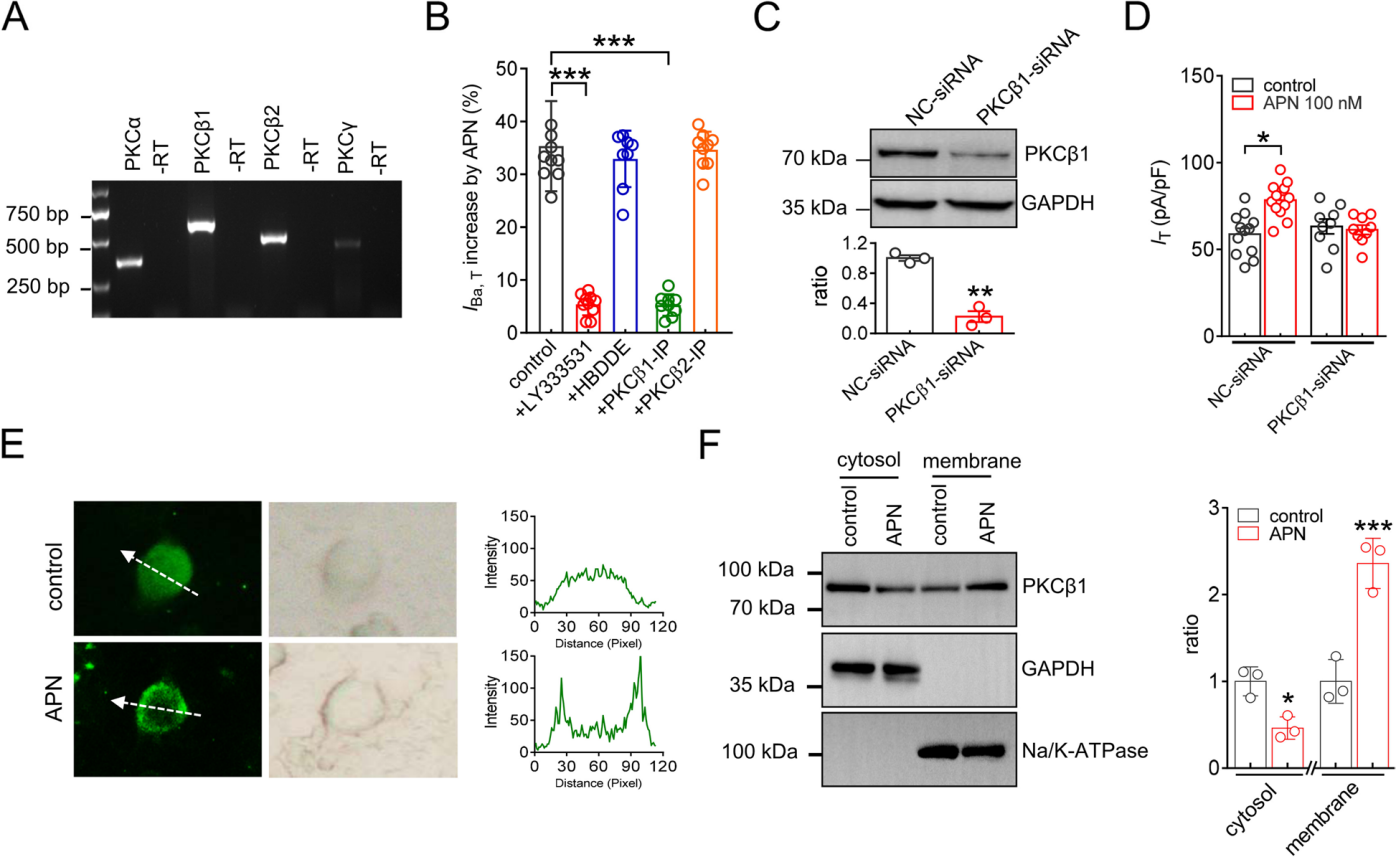
PKCβ1 is involved in the adipoR1-induced *I*_T_ response. **A** Determination of mRNAs of classic PKC isoforms (PKCα, PKCβ1, PKCβ2 and PKCγ) in the TG of mice. No signal was detected in the reactions without RT (− RT). **B** Bar graph revealing the effect of 100 nM adiponectin on *I*_T_ in the presence of LY333531 (200 nM, *n* = 10 cells), HBDDE (2 µM, *n* = 8 cells), PKCβ1 inhibitory peptide (PKCβ1-IP, 10 µM, *n* = 9 cells), or PKCβ2-IP (10 µM, *n* = 9 cells). ***p < 0.001 (*vs.* control), paired *t* test. **C** Protein abundance of PKCβ1 in TG cells treated with NC-siRNA or PKCβ1-siRNA. Representative blots of at least 3 independent experiments are shown. **p < 0.01 (*vs.* NC-siRNA), unpaired *t* test. **D** Bar graph revealing that treatment with PKCβ1-siRNA prevented the adiponectin-induced *I*_T_ increase (*n* = 9 cells). Adiponectin at 100 nM still significantly enhanced *I*_T_ in cells transduced with NC-siRNA (*n* = 12 cells). *p < 0.05 (*vs.* control + NC-siRNA), unpaired *t* test. **E** Immunofluorescence analysis of PKCβ1 translocation mediated by 100 nM adiponectin. Arrows in white indicate the line-scanned area. Data are representative of 3 independent experiments. **F** Immunoblot analysis of PKCβ1 expression in cytoplasmic and membrane fractions isolated from TG cells treated with 100 nM adiponectin. GAPDH served as a control for protein loading. α-Na^+^/K^+^ ATPase was used as an indicator for membrane contamination of cytosolic extracts. Representative blots of at least 3 independent experiments are shown. **p < 0.01 (*vs.* control), unpaired *t* test

The precise classic PKC isoform was further determined. We revealed that all four classical isoforms, including PKCα, PKCβ1, PKCβ2 and PKCγ, were endogenously expressed in the TG, with the PKCγ level being comparatively lower (Fig. 5A & Fig. S8). Although pretreating TG neurons with HBDDE (1 μM), a PKCα and PKCγ inhibitor, did not alter the adiponectin-induced *I*_T_ increase (increase 32.1% ± 5.2%, Fig. 5B), preincubation with LY333531 (200 nM), a selective inhibitor of PKCβ, abrogated the adiponectin-mediated *I*_T_ response (increase 5.3% ± 2.1%, Fig. 5B). Similar blockade of the adiponectin response was observed when a specific PKCβ1 inhibitory peptide (PKCβ1-IP) was intracellularly applied. Dialysis of TG neurons with PKCβ1-IP, but not PKCβ2-IP, prevented the adiponectin-mediated *I*_T_ response (increase 5.1% ± 2.3%, Fig. 5B). To confirm this PKCβ1-mediated response, we applied intra-TG administration of siRNA to knockdown PKCβ1 expression in TG cells. Compared to the substantial expression of PKCβ1 in the control siRNA groups, the protein abundance of PKCβ1 was dramatically decreased in TG cells treated with PKCβ1-siRNA (Fig. 5C & Fig. S9). Knockdown of PKCβ1 in TG neurons abolished the adiponectin–induced increase in *I*_T_ (increase -1.9% ± 1.5%, Fig. 5D). Moreover, PKC translocation is a typical indicator of its activation. We therefore determined the adiponectin-induced translocation of PKCβ1 from the cytosol to the plasma membrane. Immunofluorescent labeling revealed that adiponectin at 100 nM clearly induced PKCβ1 recruitment to the membrane of the soma (Fig. 5E). Consistent with this finding, adiponectin at 100 nM induced a significant increase in membrane-bound PKCβ1 and a decrease in the cytosolic fraction (Fig. 5F & Fig. S10).

### AdipoR1 selectively stimulates recombinant Cav3.2 channels

**Fig. 6:**
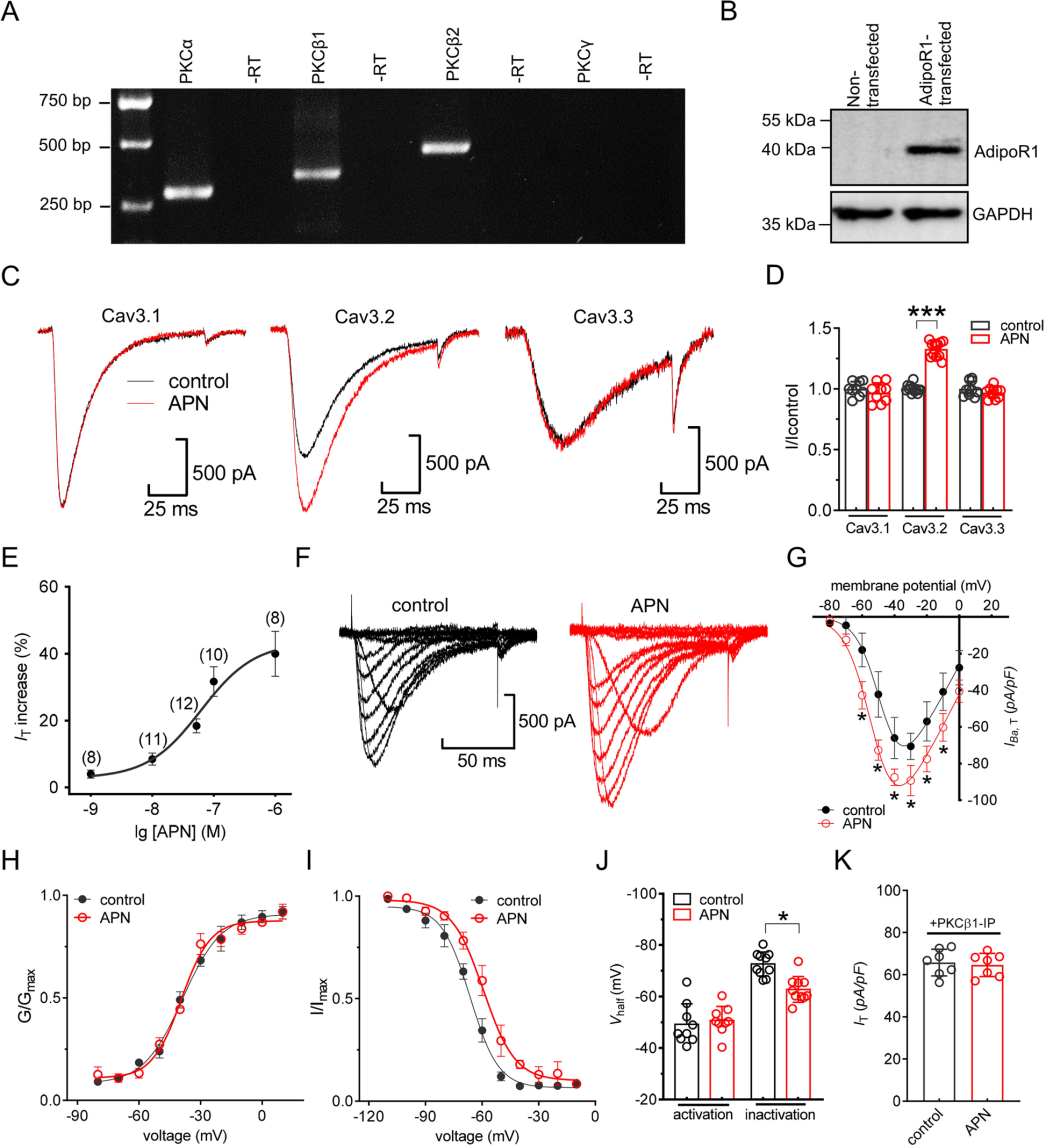
AdipoR1 selectively enhances recombinant Cav3.2 channel currents. **A** Determination of mRNAs of classic PKC isoforms (PKCα, PKCβ1, PKCβ2 and PKCγ) in HEK293 cells. No signal was detected in the reactions without RT (− RT). **B** Protein abundance of adipoR1 in HEK293 cells transiently transfected with *adipoR1* cDNA. GAPDH served as an equal loading control. Representative blots of at least 3 independent experiments are shown. **C** Representative traces revealing the effect of adiponectin (100 nM) on recombinant Cav3.1, Cav3.2 and Cav3.3 T-type channel currents. *I*_T_ was elicited by a step-pulse depolarization from a holding potential of -90 mV to -30 mV. **D** Bar graph revealing the effect of adiponectin (100 nM) on Cav3.1 (*n* = 9 cells), Cav3.2 (*n* = 12 cells) or Cav3.3 (*n* = 10 cells) channel currents indicated in Panel *D*. ***p < 0.001 (*vs.* control), paired *t* test. **E,** Dose‒response curves of adiponectin on Cav3.2 channel currents. The solid line represents the best fit with the *Hill* equation. In brackets is the number of cells recorded at each concentration of adiponectin. **F-G** Representative traces (*left*) and bar graph (*right*) revealing the effect of adiponectin (100 nM) on the current–voltage curve (*n* = 12 cells). Currents were recorded by step pulse depolarizations to potentials ranging from -80 mV to 0 mV in 10-mV increments. *p < 0.05 (*vs.* control), one-way ANOVA. **H-I** Adiponectin at 100 nM did not significantly alter the voltage-dependent activation profile of *I*_T_ (*n* = 10 cells) but shifted the steady-state inactivation curve in the depolarizing direction (*n* = 11 cells). **J** Bar chart summarizing the effect of adiponectin on the *V*_50_ of the activation or inactivation curve. *p < 0.05 (*vs.* control), unpaired *t* test. **K** Bar graph revealing the effect of adiponectin (100 nM) on recombinant Cav3.2 channel currents in cells dialyzed with PKCβ1-IP (10 μM, *n* = 7 cells)

We next examined specific interactions between Cav3 T-type channel subtypes and adipoR1. RT–PCR analysis demonstrated that both PKCα and PKCβ, including PKCβ1 and PKCβ2, were endogenously expressed in HEK293 cells, while PKCγ was not detected (Fig. 6A & Fig. S11). Examination of HEK293 cell protein lysates did not reveal the endogenous expression of adipoR1 (Fig. 6B & Fig. S12). Therefore, we transiently cotransfected cloned adipoR1 with individual α1 subunits of recombinant Cav3 channels into HEK293 cells. Immunoblot analysis of transfected HEK293 cells showed a predominant band (~ 43 kDa) after adipoR1 overexpression (Fig. 6B). Activation of adipoR1 by 100 nM adiponectin had a stimulatory effect on Cav3.2 channels but comparatively no effects on either Cav3.1 or Cav3.3 currents (Fig. 6C and D). Testing the effects of varying concentrations of adiponectin revealed that the stimulatory effect is dose-dependent (Fig. 6E). Further examination of the biophysical characteristics of Cav3.2 channels indicated that adiponectin at 100 nM caused the current–voltage curve to shift down and enhanced the currents from -66.3 ± 11.7 pA/pF to -87.3 ± 4.2 pA/pF at -40 mV (Fig. 6F and G). Application of 100 nM adiponectin did not affect the property of voltage-dependent activation (Fig. 6H and J) but produced a depolarizing shift (~ 7.9 mV) of the steady-state inactivation curve (Fig. 6I and J). In addition, dialysis of cells with PKCβ1-IP (10 µM) completely prevented the adiponectin-induced increase in Cav3.2 channel currents (increase -2.2% ± 1.7%, Fig. 6K).

### Adiponectin increases TG neuronal excitability via Cav3.2 channels

**Fig. 7:**
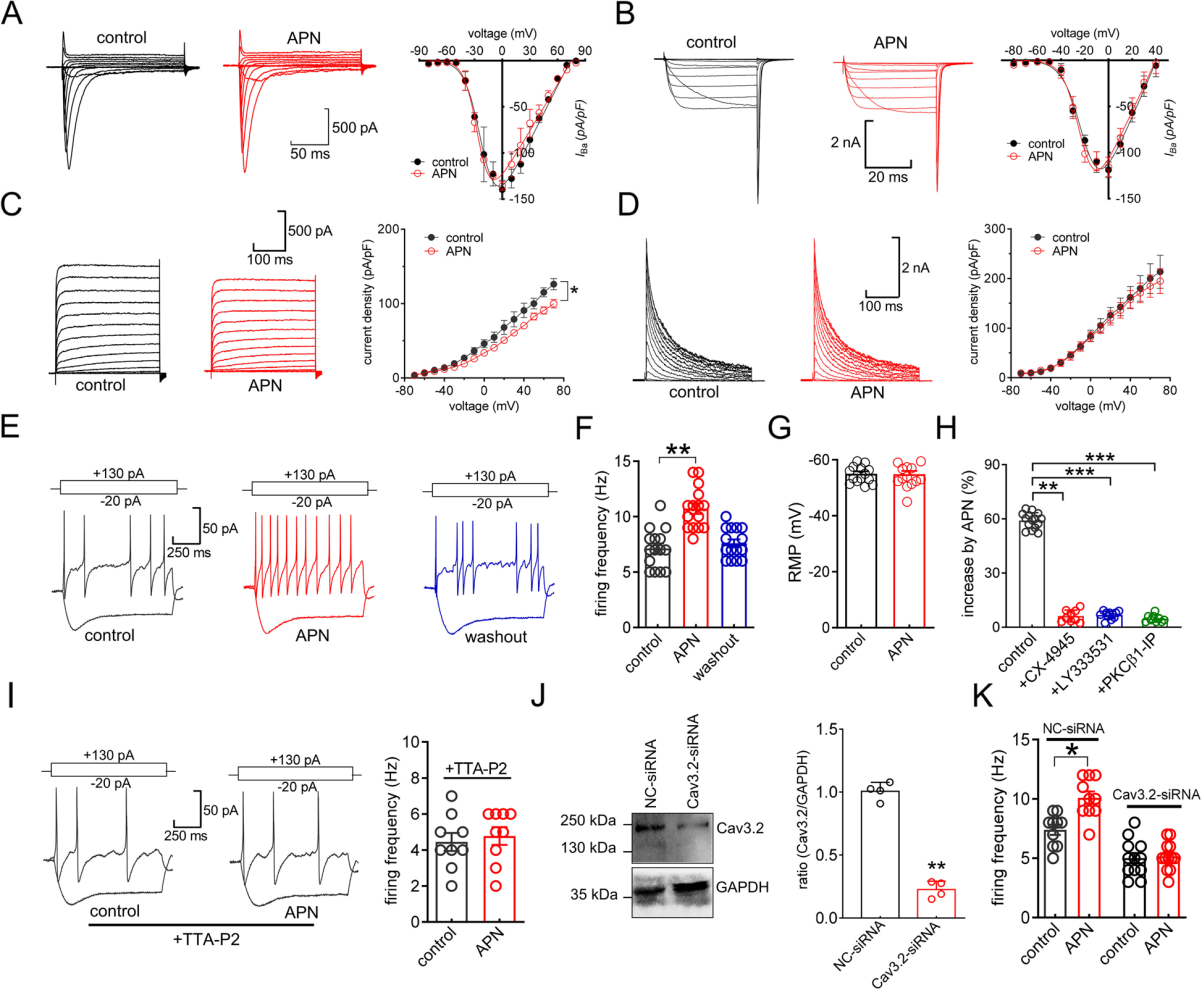
Adiponectin increases membrane excitability in TG neurons. **A-D** Representative traces (*left*) and bar chart (*right*) showing the effect of 100 nM adiponectin on Nav currents (*I*_Na_, *n* = 8 cells,* A*), high-voltage activated (HVA) Ca^2+^ currents (*n* = 9 cells,* B*), sustained delayed-rectifier K^+^ currents (*I*_DR_, *n* = 12 cells, *C*), and transient outward A-type K^+^ currents (*I*_A_, *n* = 12 cells, *D*). TG neurons were held at -90 mV, and *I*_Na_ was stimulated with a series of depolarizing pulses ranging between -80 and + 80 mV. HVA Ca^2+^ currents were elicited from the holding potential of -60 mV with depolarizing pulses ranging between -80 and + 40 mV. To obtain the current–voltage relationship of *I*_A_ and *I*_DR_, neurons were held at -80 mV and stimulated with step depolarizing pulses ranging from -70 tp + 70 mV in 10-mV increments. *p < 0.05 (*vs.* control), one-way ANOVA. **E–F** Representative traces (*E*) and bar chart (*F*) showing that adiponectin at 100 nM markedly increased the rate of action potential (AP) firing (*n* = 15 cells). *Insets* depicted at top represent the protocols of current injection. **p < 0.01 (*vs.* control), paired *t* test. **G** Effect of 100 nM adiponectin on the resting membrane potential of AP firing (*n* = 15 cells). **H** Bar chart showing that pretreatment of neurons with CK-4945 (10 μM,* n* = 9 cells), LY333531 (200 nM, *n* = 11 cells) or PKCβ2-IP (10 μM,* n* = 11 cells) abrogated the adiponectin-induced increase in the rate of AP firing. **p < 0.01, ***p < 0.001 (*vs.* control), paired *t* test. **I** Representative traces (*left*) and bar chart (*right*) revealing that pretreatment with TTA-P2 (3 μM) prevented the 100 nM adiponectin-induced increase in the rate of AP firing (*n* = 9 cells). **J** Protein abundance of Cav3.2 in NC-siRNA- or Cav3.2-siRNA-treated groups. Representative blots of at least 3 independent experiments are shown. **p < 0.05 (*vs.* NC-siRNA), unpaired *t* test. **K** Bar chart showing that treatment with Cav3.2-siRNA (*n* = 12 cells), but not NC-siRNA (*n* = 10 cells), prevented the 100 nM adiponectin-induced increase in the rate of AP firing. *p < 0.05 (*vs.* control + NC-siRNA), unpaired *t* test

We further tested whether adiponectin affects the membrane excitability of TG neurons to determine the functional roles of adipoR1-mediated modulation of Cav3.2 channels. Initial examination revealed that the voltage-gated Na^+^ currents (*I*_Na_) were unaffected when 100 nM adiponectin was applied (Fig. 7A). It has been demonstrated that adiponectin might facilitate L-type Ca^2+^ channels in primary cultured rat pituitary cells [50]. Thus, we applied 5 μM nifedipine to the external solution to block L-type channels and found that adiponectin at 100 nM had no significant effects on the remaining high voltage-activated Ca^2+^ currents (Fig. 7B). Moreover, the peak amplitude of the sustained delayed rectifier K^+^ currents was also reduced by adiponectin at 100 nM (*I*_DR_, Fig. 7C), while the transient outward A-type K^+^ currents (*I*_A_) remained unaffected (Fig. 7D). Therefore, in an extracellular solution containing 5 μM nifedipine to block L-type channels and 5 mM tetraethylammonium (TEA) to block *I*_DR_ channels, adiponectin at 100 nM induced a significant increase in the rate of action potential (AP) firing by 63.3 ± 5.7% (Fig. 7E and F), while other characteristics of membrane excitability, such as the resting membrane potential (Fig. 7G), remained unchanged. Pretreatment with the CK2 inhibitor CX-4945 completely abolished the increased AP firing rate induced by adiponectin (Fig. 7H). Similar abolishment was obtained when TG neurons were dialyzed with PKCβ1-IP or pretreated with the PKCβ inhibitor LY333531 (Fig. 7H). Moreover, we determined whether the increased neuronal excitability mediated by adiponectin was reliant on the stimulation of T-type channels. Pretreatment of cells with TTA-P2 (3 μM) abrogated the adiponectin-induced increase in the rate of AP firing (Fig. 7I). Next, we applied a chemically modified siRNA knockdown approach to investigate the involvement of Cav3.2 in adipoR1-mediated neuronal hyperexcitability. Intra-TG injection of Cav3.2-siRNA caused a considerable downregulation of Cav3.2 protein expression (Fig. 7J & Fig. S13). The adiponectin-mediated increase in the AP firing rate in TG neurons was completely eliminated by knockdown of Cav3.2 (Fig. 7K).

### Cav3.2 contributes to adiponectin-mediated pain hypersensitivity

**Fig. 8:**
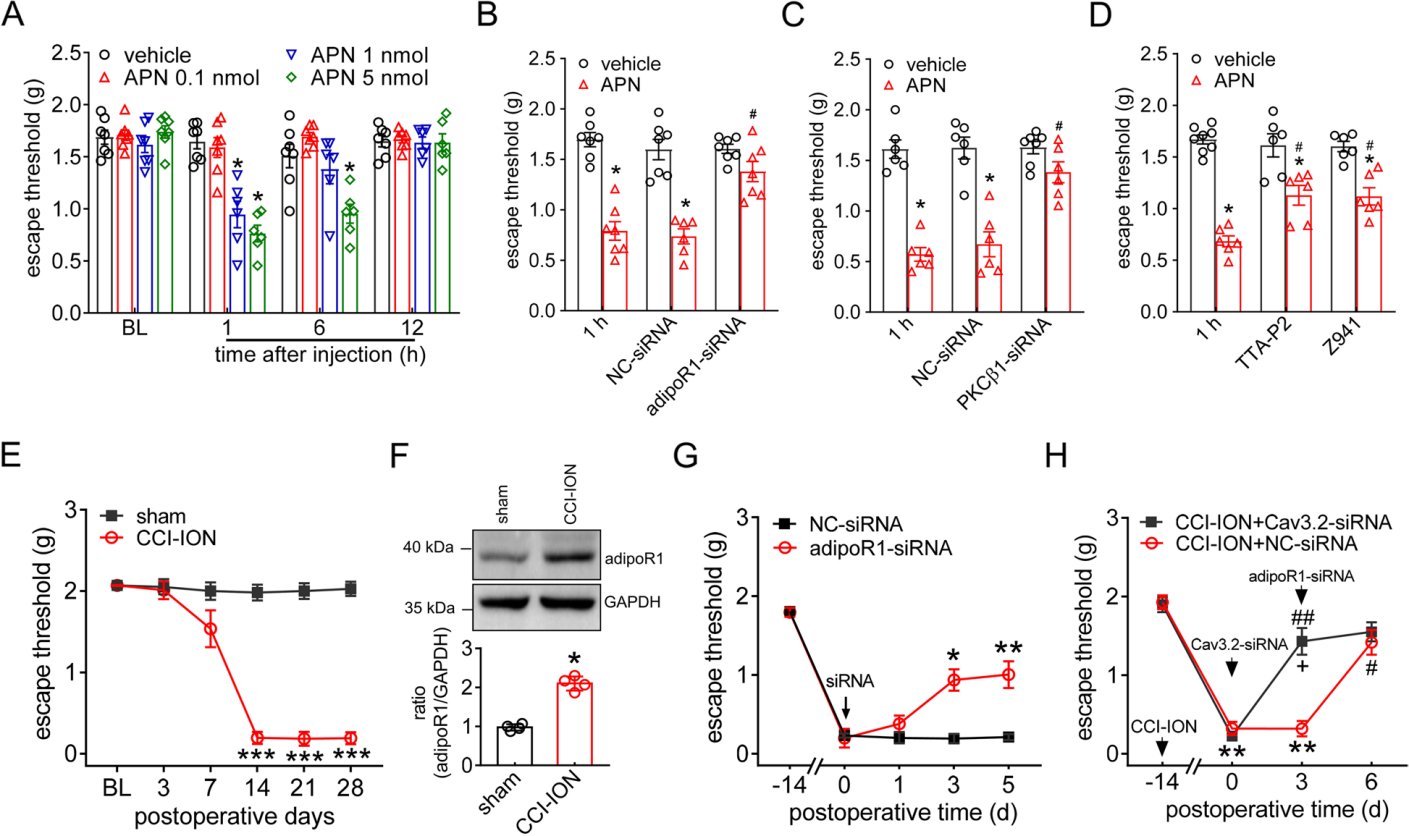
Peripheral adipoR1 participated in mechanical pain hypersensitivity. **A** Escape threshold after intra-TG injection of vehicle or adiponectin at 0.1 nmol, 1 nmol or 5 nmol. *p < 0.05 (*vs.* vehicle) at the corresponding time point, two-way ANOVA. **B**, **C** Administration of adipoR1-siRNA (*B*) or PKCβ1-siRNA (*C*) prevented adiponectin-induced mechanical hypersensitivity. *p < 0.05 (*vs.* vehicle); ^#^*p* < 0.05 (*vs.* APN) in NC-siRNA-treated groups, two-way ANOVA. **D** Pretreatment with TTA-P2 (1 nmol) or Z941 (0.5 nmol) attenuated adiponectin (1 nmol)-induced mechanical hypersensitivity. *p < 0.05 (*vs.* vehicle), ^#^*p* < 0.05 (*vs.* APN) at the 1-h time point, two-way ANOVA. **E** Escape threshold to mechanical stimuli in the sham- or CCI-ION-operated groups. ***p < 0.01 (*vs.* sham) at the corresponding time point, two-way ANOVA. **F** Protein abundance of adipoR1 in TGs 14 days following CCI-ION or sham surgery. *p < 0.05 (*vs.* sham), unpaired *t* test. Representative blots of at least 3 independent experiments are shown. **G** Intra-TG administration of adipoR1-siRNA 14 days after CCI-ION significantly attenuated mechanical hypersensitivity in CCI-ION mice. *p < 0.05 and **p < 0.01 (*vs.* NC-siRNA), two-way ANOVA. **H** Effect of Cav3.2-siRNA *vs.* NC-siRNA (Day 0) on adipoR1-siRNA (intra-TG injection on Day 3)-induced alleviation of mechanical allodynia in CCI-ION mice. Intra-TG injection of adipoR1-siRNA did not have additive effects to Cav3.2-siRNA on mechanical allodynia in CCI-ION mice. ** *p* < 0.01 (*vs.* CCI-ION) at -14 days, ^#^ *p* < 0.05 and ^##^ *p* < 0.01 (*vs.* NC-siRNA) at the 3-day point in CCI-ION mice, ^+^ *p* < 0.05 (*vs.* Cav3.2-siRNA) at the 0-day point in CCI-ION mice, two-way ANOVA

Furthermore, we examined whether adipoR1-mediated Cav3.2 signaling contributed to animal nociceptive behaviors. Intra-TG administration of adiponectin markedly enhanced acute pain sensitivity to mechanical stimuli, with 5 nmol adiponectin resulting in stronger effects than 1 nmol, while 0.1 nmol adiponectin did not elicit any effect (Fig. 8A). The effect recovered 6 h after adiponectin application. AdipoR1-siRNA delivery into the TG prevented the mechanical hypersensitivity induced by adiponectin, while intra-TG delivery of control siRNA had no effects (Fig. 8B). Similarly, preintra-TG injection of PKCβ1-siRNA eliminated adiponectin-induced hypersensitivity to mechanical pain (Fig. 8C). TTA-P2 was used to further determine the participation of T-type channels in the adiponectin-mediated behavior response (Fig. 8D). Delivery of TTA-P2 into the TG had no effect on the escape threshold; however, prior intra-TG injection of TTA-P2 attenuated adiponectin-induced mechanical hypersensitivity (Fig. 8D). Similar findings were obtained by applying Z941, another particular blocker of T-type channels [51]. Furthermore, the potential contribution of adiponectin to chronic neuropathic pain was also investigated. A mouse model of trigeminal neuralgia was induced by chronic constriction injury to the infraorbital nerve (CCI-ION). Compared to sham surgery, mice exhibited a significant reduction in the escape threshold to mechanical stimuli on days 14, 21, and 28 after CCI-ION operation (Fig. 8E). At Day 14, when mechanical allodynia peaked, TGs showed a robustly concomitant increase in adipoR1 protein abundance (Fig. 8F & Fig. S14). Thus, the role of adipoR1 inhibition in nerve injury-induced mechanical allodynia was determined. In contrast to NC-siRNA treatment, intra-TG administration of adipoR1-siRNA significantly alleviated mechanical allodynia on Day 14 after CCI-ION (Fig. 8G). To validate the role of Cav3.2 channels as pivotal targets for pain relief of adiponectin signaling in trigeminal-mediated neuropathic pain, we applied chemically modified Cav3.2-siRNA (or control siRNA) by intra-TG injection and found that administration of Cav3.2-siRNA induced marked alleviation of mechanical allodynia in CCI-ION-treated mice, while treatment with control siRNA did not elicit any improvement in escape threshold (Fig. 8H). Mechanical sensitivity in Cav3.2-siRNA-treated CCI-ION mice was measured following the injection of adipoR1-siRNA, and the results revealed that adipoR1-siRNA had no appreciably additive effect to Cav3.2 siRNA (Fig. 8H). In contrast, mice that received injections of control siRNA of Cav3.2 responded to adipoR1-siRNA, which is similar to those that did not receive siRNA injection in that administration of adipoR1-siRNA caused a marked increase in the escape threshold (Fig. 8G). These findings imply that Cav3.2 channels participate in adipoR1-mediated pain hypersensitivity in trigeminal neuralgia.

## Discussion

**Fig. 9:**
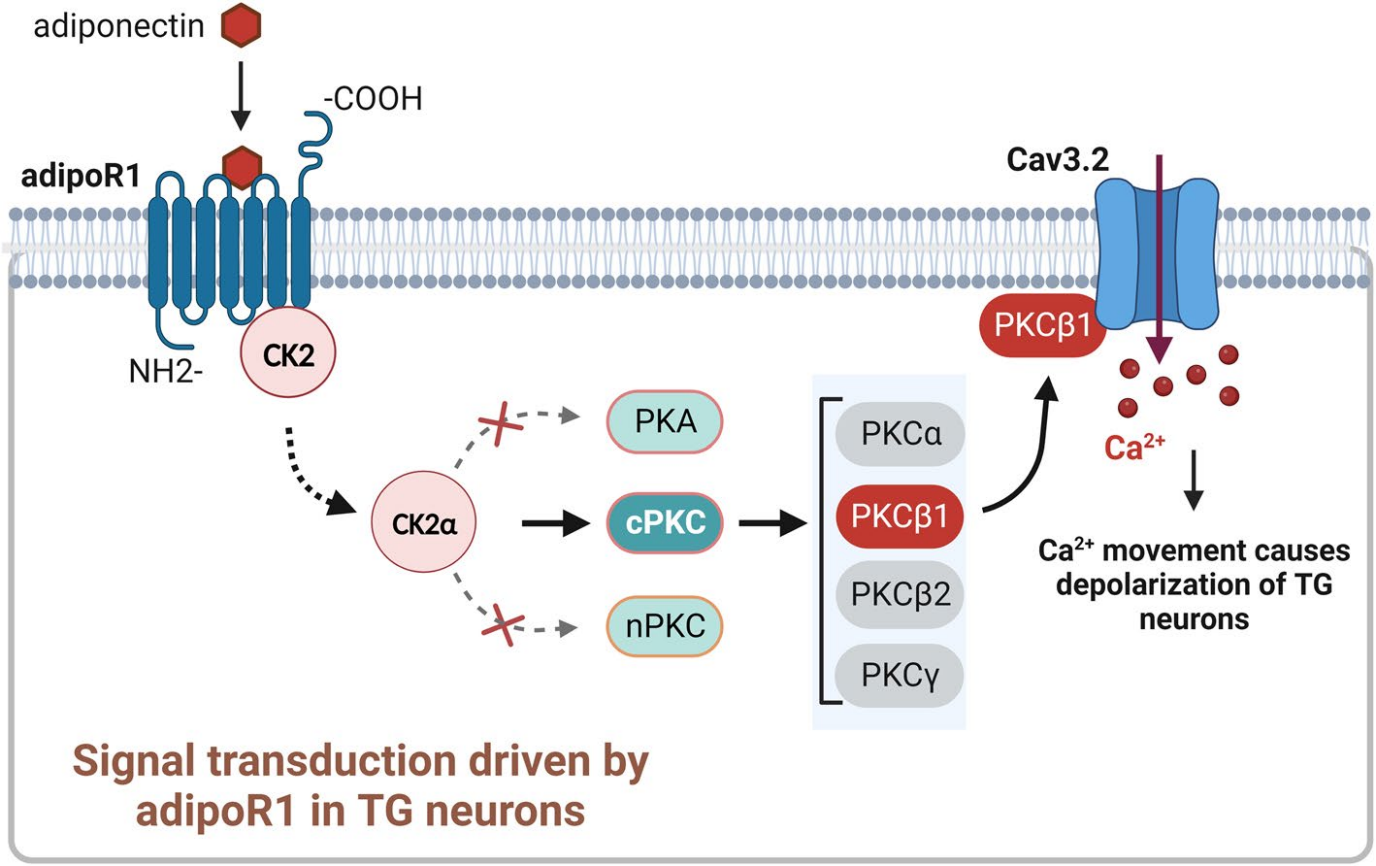
Illustration of proposed mechanisms of adipoR1 signaling on Cav3.2 channels. Adiponectin acts through the G protein-independent adipoR1, leading to the activation of protein kinase CK2α subunits. CK2α stimulates the downstream conventional PKCβ1, which selectively modulates Cav3.2 channel activity, resulting in an *I*_T_ increase. The signaling cascade mediated by adipoR1 contributes to TG neuronal hyperexcitability and nociceptive behaviors of adiponectin. Importantly, adipoR1 was upregulated in the injured TG, and blockade of Cav3.2 attenuated adipoR1-mediated pain hypersensitivity in CCI-ION-induced neuropathic pain behaviors. Neither PKA, CaMKII nor novel PKC isoforms are involved in the adiponectin-mediated *I*_T_ response. Nevertheless, whether PKCβ1 directly phosphorylates Cav3.2 channels or acts via some intermediate signaling molecules needs to be further explored

In this study, we provided mechanistic insights into a critical role of adiponectin in regulating Cav3.2 channels in TG neurons. The results demonstrated that the adiponectin effect was mediated by adipoR1 coupling to CK2α and triggering the activation of downstream PKCβ1 signaling (see Fig. 9 for an illustration of the proposed mechanism). The adipoR1-mediated *I*_T_ response contributes to increased TG neuronal excitability and nociceptive behaviors. Targeting adipoR1-mediated signaling offers a novel therapeutic strategy/target for trigeminal-mediated pain syndrome.

AdipoRs belong to a novel group of membrane receptors predicted to have seven transmembrane domains but that are structurally and topologically distinct from GPCRs [6]. Distinct from GPCRs, which normally stimulate G protein and downstream signaling cascades, adiponectin/adipoR initiates signal transduction events in a manner independent of G proteins [40]. In this study, dialysis with GDP-β-S did not affect the adiponectin-induced *I*_T_ response. Several intracellular binding partners of the adipoR N-terminus, such as RACK1 and ERp46, have been identified and shown to control receptor signaling in a tissue-specific manner [52]. For instance, in transfected tsA-201 cells, one study identified that overexpressed RACK1 interacts with the C-terminus of recombinant Cav3.2 channels [53]. However, coimmunoprecipitation analysis of mouse TGs in the present study did not show the formation of a molecular protein complex between adipoR1 and RACK1, although both were endogenously detected. Interestingly, further examination identified the protein kinase CK2α, one isoform of the catalytic subunit of serine/threonine kinase CK2, as the interaction partner of adipoR1 in mouse TGs. CK2α mediates the adipoR1-mediated T-type channel response since 1) pretreating cells with CK2 inhibitors abrogated the adiponectin-induced T-type channel response and 2) knockdown of CK2α, but not RACK1, prevented the adiponectin-induced *I*_T_ increase. CK2 can stimulate PKA to phosphorylate various downstream molecular targets [46], and accumulated evidence suggests divergent roles of PKA in modulating *I*_T_ [47]. For instance, PKA activation by forskolin in ventricular myocytes [54] was shown to stimulate T-type channels. Similarly, T-type channel currents recorded from recombinant Cav3 channels in *Xenopus oocytes* might be enhanced by PKA inducers [55]. This result was reproducible in rat glomerulosa cells in which inhibition of PKA activity prevented the serotonin type 7 receptor-induced *I*_T_ increase [47]. In contrast, in retinal horizontal cells, dopamine-induced *I*_T_ inhibition was prevented by PKA inhibitors [56], and *I*_T_ inhibition induced by adrenaline in newt olfactory receptor cells was mimicked by the intracellular application of the catalytic subunit of PKA [57]. Intriguingly, studies have also suggested biphasic effects or even no effect of PKA activation on *I*_T_ [47]. Nevertheless, the possibility that the adiponectin-induced *I*_T_ increase was due to CK2α-PKA activation can be ruled out in the present study, suggesting that alternative non-PKA-mediated signaling is involved.

PKC is a pivotal regulator of T-type channels [47]. Studies investigating T-type channel modulation via PKC-dependent pathways have revealed conflicting conclusions, in that *I*_T_ has been reported to be either upregulated or downregulated [47, 58]. From our results, we demonstrated that CK2α-dependent PKC participated in the adipoR1-mediated *I*_T_ increase in TG neurons. This is supported by earlier findings in neonatal rat cardiomyocytes, which showed that the PKC activator phorbol myristate acetate (PMA) enhanced *I*_T_, whereas an inactive counterpart had no effect [59]. Similarly, PKC inhibition eliminated the increase in *I*_T_ induced by insulin-like growth factor-1 (IGF-1) in dorsal root ganglion (DRG) neurons [32]. In contrast, suppression of *I*_T_ induced by M3 muscarinic receptor activation was abolished by PKC antagonists [60]. Similar findings were observed for the exogenously expressed Cav3 channel subtypes with neurokinin 1 receptor in HEK293 cells, which resulted in a PKC-dependent decrease in Cav3.2 channel currents [48]. Intriguingly, these results were not replicated in Cav3.2 channels reconstituted in *Xenopus oocytes* [49]. One of the explanations to the discrepancies is that PKC-interacting proteins, such as those for Cav2.2 channels, can be one of these factors [61]. Known PKC-interacting proteins regulate the activity of specific PKC isoforms conferring cell-type-specific functional regulation [62]. Additional possibilities include the fact that splice variant-specific regulation of Cav3.2 occurs and that T-type channels are vulnerable to considerable alternative splicing [63]. Furthermore, we cannot rule out the possibility that the adiponectin-mediated response was mediated by an intermediary protein that was phosphorylated by a different PKC isoform. In addition, non-selective PKC activation by PMA increases T-type channel currents in tsA-201 cells or Chinese hamster ovary (CHO) cell line stably expressing Ca_V_3.2 channels [55]. Interestingly, this effect was shown to occur only at 37 °C, but not at room temperature. The temperature dependence suggested by the authors might involve kinase translocation, which could be impaired at room temperature [55]. However, stimulation of PKC might lead to its rapid translocation from the cytoplasm to the plasma membrane at room temperature, e.g., treatment of sensory neurons by IGF-1 resulted in the translocation of PKCα from the cytosol to the membrane fraction [32]. In addition, low-frequency stimulation that induces long-term depression in CA1 hippocampal slices also causes conventional PKC isoforms to translocate to the plasma membrane [64]. This transitory nature of the translocation supports a similar potential type of involvement of TG PKCβ1 in adiponectin responses.

T-type channels are unique among voltage-gated Ca^2+^ channels in that they function near the resting membrane potential of neurons [65]. This low activation threshold property in peripheral nociceptors results in the increased neurotransmission of sensory neurons and, in particular, increased pain perception [66, 67]. Recent evidence has indicated that modulation of peripheral Cav3.2 channels influences nociceptive inputs and that inhibition of T-type channels results in marked antinociceptive effects in a variety of pain models [68, 69]. In the present study, we demonstrated that activation of adipoR1 enhanced TG neuronal excitability and induced mechanical pain hypersensitivity; the effects were prevented by the inhibition of PKCβ1 or T-type channels. Furthermore, the blockade of adipoR1 signaling alleviated mechanical allodynia in nerve injury-induced neuropathic pain, which was attenuated by Cav3.2-siRNA. As such, the PKC1-mediated stimulation of Cav3.2 channels was involved in the nociceptive effects of adipoR1 activation. In line with this, the *Adipor1* gene has been shown to be potentially associated with the severity of postoperative pain [9]. Importantly, these findings are supported by clinical evidence showing that chronic headache sufferers have higher adiponectin levels in the serum [12, 13]. Further support of this finding is that adiponectin enhances the membrane excitability of rat paraventricular nucleus neurons [70]. Interestingly, contradictory results in some previous studies also revealed an anti-nociceptive effect of adiponectin. For instance, intrathecal injection of adiponectin alleviated carrageenan-induced inflammatory pain in rats [10], and obese rats with inflammatory hyperalgesia had decreased adiponectin levels in the spinal cord [71]. Although the discrepancies have yet to be clarified, the adiponectin-mediated analgesic effects might involve central rather than peripheral actions of adiponectin. In addition, it should also be noted that other than adipoR1 being expressed only in the TG, the potential mechanisms of adipoR2 expressed in the spinal cord also contribute to rat inflammatory pain [11]. Of note, this variability may be influenced by intraspecies variations and variations in the ages of the animals utilized in the various experiments [72, 73]. Interestingly, specific pathways in the spinal microglia or DRG macrophages were involved in the sexual dimorphism of neuropathic pain [74, 75]. Sexual dimorphism seems to be limited to microglial or macrophages, since the inhibition of pain-related signaling in neurons and astrocytes produced similar analgesia in both sexes [76, 77]. Given that adipoR1 are expressed exclusively in the TG neurons, it accounts for the consistency of male and female mice pain symptoms observed in the present study. Nonetheless, the significance and underlying mechanism for the role of gender differences in adipoR1-mediated pain regulation warrants further investigation.

## Conclusion

In summary, we present new insights and dissect the molecular components that underlie the effect of adiponectin on Cav3.2 channels. Our study provides evidence that stimulation of adipoR1 in TG neurons enhances Cav3.2 channel currents through CK2α-dependent PKCβ1 signaling. This mechanism is proposed to enhance neuronal excitability and contribute to pain hypersensitivity. Knowledge of the adipoR1-mediated CK2α-PKCβ1-Cav3.2 cascade in peripheral sensory neurons may pave the way for developing potential therapeutic targets in clinical treatment of pain disorders such as trigeminal neuralgia.

### Supplementary Information

**Additional file 1: Table S1.** Primers used for RT-PCR analysis of PKC isoforms in mouse TGs. **Table S2.** Primers used for RT-PCR analysis of PKC isoforms in HEK293 cells. **Fig. S1.** Protein expression of adipoR1 and adipoR2 in mouse TGs. **Fig. S2.** Knockdown of adipoR1 in TGs. **Fig. S3.** Co-immunoprecipitation analysis of the association of RACK1 with adipoR1 in mouse TGs. **Fig. S4.** Knockdown of RACK1 in mouse TGs. **Fig. S5.** Association of CK2 alpha with the adipoR1 in mouse TGs. **Fig. S6.** Knockdown of CK2α in mouse TGs. **Fig. S7.** Protein expression of p-CaMKII and t-CaMKII in mouse TGs. **Fig. S8.** RT-PCR analysis of mRNAs of classic PKC isoforms (PKCα, PKCβ1, PKCβ2 and PKCγ) in mouse TGs. **Fig. S9.** Knockdown of PKCβ1 in mouse TGs. **Fig. S10.** Immunoblot analysis of PKCβ1 expression in cytoplasmic and membrane fractions. **Fig. S11.** RT-PCR analysis of mRNAs of classic PKC isoforms (PKCα, PKCβ1, PKCβ2 and PKCγ) in HEK293 cells. **Fig. S12.** Protein expression of adipoR1 in HEK293 cells transfected with ADIPOR1 cDNA. **Fig. S13.** The increased expression level of Cav3.2 induced by CCI-ION was attenuated by intra-TG injection of Cav3.2-siRNA. **Fig. S14.** Protein expression of adipoR1 in mouse TGs after CCI-ION.

## Data Availability

All data and materials generated in this study are available upon request.

## Abbreviations

AdipoR1: Adiponectin receptor 1
TG: Trigeminal ganglion
*I*_T_: T-type channel currents
CGRP: Calcitonin gene-related peptide
PKA: Protein kinase A
PKCβ1: Protein kinase C beta1
CK2α: Casein kinase II alpha-subunits
RACK1: Receptor for Activated C Kinase 1
CCI-ION: Chronic constriction injury of the infraorbital nerve
