# Supplementary material for: Adiponectin receptor 1-mediated stimulation of Cav3.2 channels in trigeminal ganglion neurons induces nociceptive behaviors in mice
Source: J Headache Pain. 2023 Aug 25;24(1):117. doi: 10.1186/s10194-023-01658-2 (PMC10463856; doi:10.1186/s10194-023-01658-2)
Supplement: Supplementary file 1 — Additional file 1: Table S1. Primers used for RT-PCR analysis of PKC isoforms in mouse TGs. Table S2. Primers used for RT-PCR analysis of PKC isoforms in HEK293 cells. Fig. S1. Protein expression of adipoR1 and adipoR2 in mouse TGs. Fig. S2. Knockdown of adipoR1 in TGs. Fig. S3. Co-immunoprecipitation analysis of the association of RACK1 with adipoR1 in mouse TGs. Fig. S4. Knockdown of RACK1 in mouse TGs. Fig. S5. Association of CK2 alpha with the adipoR1 in mouse TGs. Fig. S6. Knockdown of CK2α in mouse TGs. Fig. S7. Protein expression of p-CaMKII and t-CaMKII in mouse TGs. Fig. S8. RT-PCR analysis of mRNAs of classic PKC isoforms (PKCα, PKCβ1, PKCβ2 and PKCγ) in mouse TGs. Fig. S9. Knockdown of PKCβ1 in mouse TGs. Fig. S10. Immunoblot analysis of PKCβ1 expression in cytoplasmic and membrane fractions. Fig. S11. RT-PCR analysis of mRNAs of classic PKC isoforms (PKCα, PKCβ1, PKCβ2 and PKCγ) in HEK293 cells. Fig. S12. Protein expression of adipoR1 in HEK293 cells transfected with ADIPOR1 cDNA. Fig. S13. The increased expression level of Cav3.2 induced by CCI-ION was attenuated by intra-TG injection of Cav3.2-siRNA. Fig. S14. Protein expression of adipoR1 in mouse TGs after CCI-ION. [file 10194_2023_1658_MOESM1_ESM.pdf]

**Supporting Information**

# Adiponectin receptor 1-mediated stimulation of Cav3.2 channels in trigeminal ganglion neurons induces nociceptive behaviors in mice

Yuan Zhang [1, 4, #, *], Yuan Wei [2, #], Tingting Zheng [1, #], Yu Tao [2], Yufang Sun [2, 4], Dongsheng Jiang [3], Jin Tao [2, 4, *]

[1] Clinical Research Center of Neurological Disease & Department of Geriatrics, The Second Affiliated Hospital of Soochow University, Suzhou 215004, P.R. China;

[2] Department of Physiology and Neurobiology & Centre for Ion Channelopathy, Suzhou Medical College of Soochow University, Suzhou 215123, P.R. China;

[3] Institute of Regenerative Biology and Medicine, Helmholtz Zentrum München, Munich 81377, Germany;

[4] Jiangsu Key Laboratory of Neuropsychiatric Diseases, Soochow University, Suzhou 215123, P.R. China.

[#] These authors contributed equally to this work.

***To whom correspondence should be addressed:**

Dr. Yuan Zhang, Clinical Research Center of Neurological Disease & Department of Geriatrics, The Second Affiliated Hospital of Soochow University, 1055 San-Xiang Road, Suzhou 215004, P.R. China. E-mail: yuanzhang@suda.edu.cn

Dr. Jin Tao, Department of Physiology and Neurobiology & Centre for Ion Channelopathy, Suzhou Medical College of Soochow University, 199 Ren-Ai Road, Suzhou 215123, P.R. China. E-mail: taoj@suda.edu.cn

**This PDF file includes: SI Tables S1 to S2**

**SI Figures S1 to S14**

**SI Tables S1 to S2**

Table S1: Primers used for RT-PCR analysis of PKC isoforms in mouse TGs

| Gene | Primers for RT-PCR | Size (bp) |
|---|---|---|
| PKCα | *F:* ACAACCTGGACAGAGTGAA | 354 |
| | *R:* TCTCGGCTGCGTAGAAT | |
| PKCβ1 | *F:* AGAGCAAGGGCATTATT | 597 |
| | *R:* CGAATTCTGATTGGTCAA | |
| PKCβ2 | *F:* ACATTCTGTGGCACTCCG | 476 |
| | *R:* GCAAACTCATTTTGGTCC | |
| PKCγ | *F:* GGCTCCGACGAACTCTAT | 417 |
| | *R:* GGACCCAGGGAAGACATT | |

Table S2: Primers used for RT-PCR analysis of PKC isoforms in HEK293 cells

| Gene | Primers for RT-PCR | Size (bp) |
|---|---|---|
| PKCα | *F:* GGCGTCCTGTTGTATGAAATGCTTG | 275 |
| | *R:* GGCTTGAATGGTGGCTGGATCTC | |
| PKCβ1 | *F:* ATTCACCGCCCGCTTCTTCAAG | 340 |
| | *R:* ACACAGGCTGGGAACATTCATCAC | |
| PKCβ2 | *F:* CCTGCTGTATGAAATGTTG | 433 |
| | *R:* GCTCTTGACTTCGGGTT | |
| PKCγ | *F:* AATTGTATGAGCGGGTGCGGATG | 351 |
| | *R:* GGGTCTGGAAGGTGGAGTGGAG | |

**SI Figures S1 to S14**

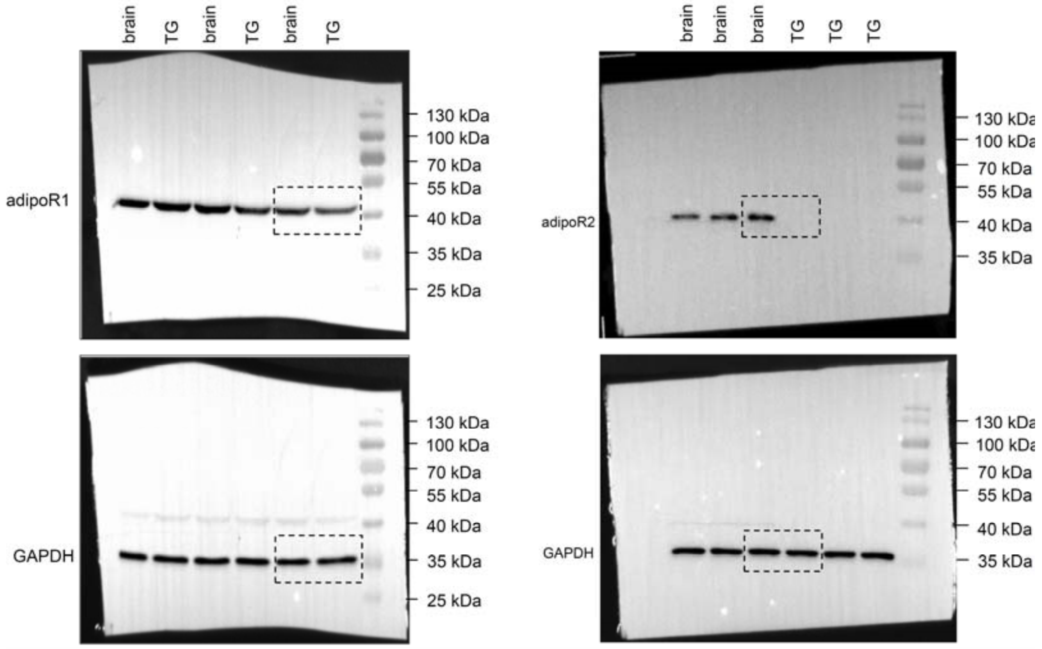


**Fig. S1: Protein expression of adipoR1 and adipoR2 in mouse TGs.** Shown are the full-length pictures of the blots for adipoR1 (*left panel*) and adipoR2 (*right panel*) respectively presented in Fig. 2A and Fig. 2B. The blots shown are representative of at least three experiments.

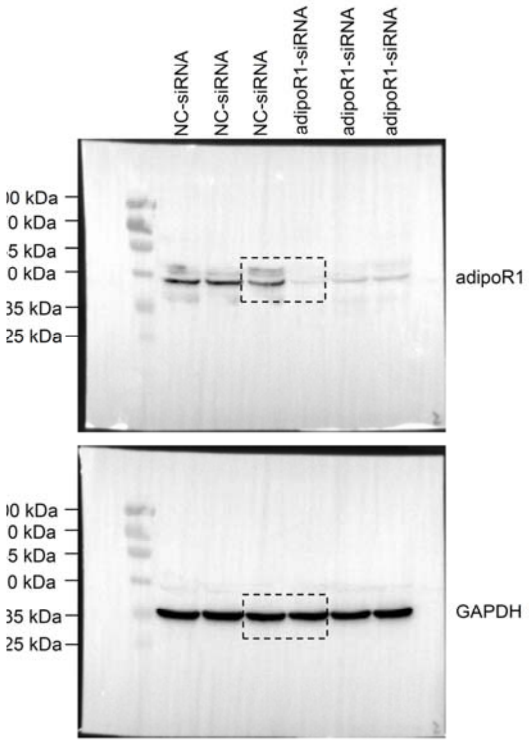


**Fig. S2: Knockdown of adipoR1 in TGs.** Shown are the full-length pictures of the blots presented in Fig. 2D. The protein abundance of adipoR1 was measured using immunoblot analysis in negative control siRNA (NC-siRNA) and adipoR1 siRNA-treated (adipoR1-siRNA) groups. The blots shown are representative of at least three experiments.

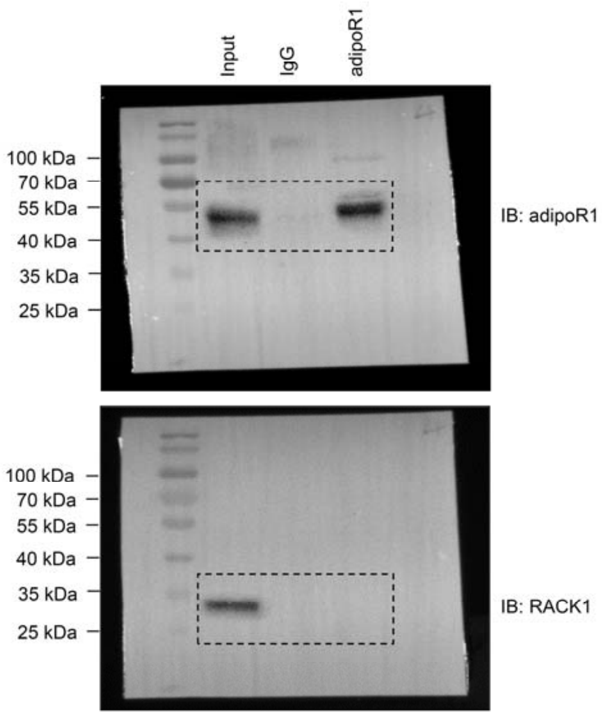


**Fig. S3: Co-immunoprecipitation analysis of the association of RACK1 with adipoR1 in mouse TGs.** Shown are the full-length pictures of the blots presented in Fig. 3B. The blots shown are representative of at least three experiments.

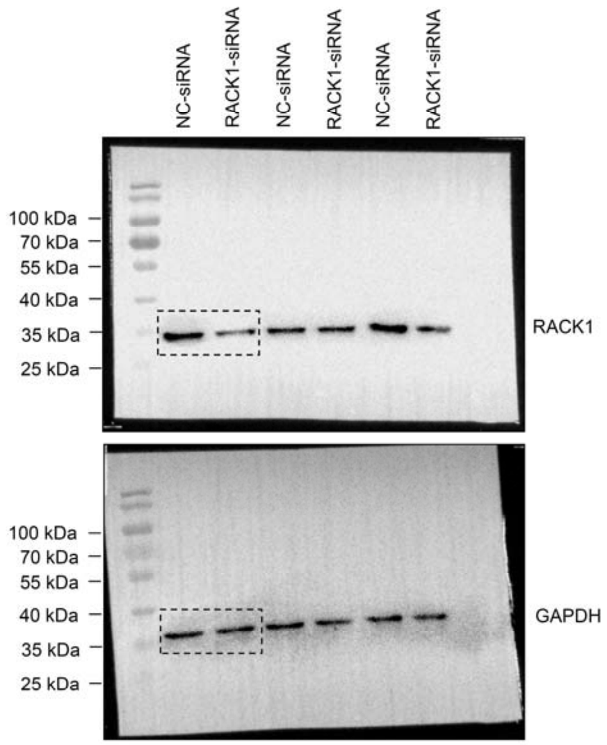


**Fig. S4: Knockdown of RACK1 in mouse TGs.** Shown are the full-length pictures of the blots presented in Fig. 3C. The protein abundance of RACK1 was measured using immunoblot analysis in negative control siRNA (NC-siRNA) and RACK1 siRNA-treated (RACK1-siRNA) groups. The blots shown are representative of at least three experiments.

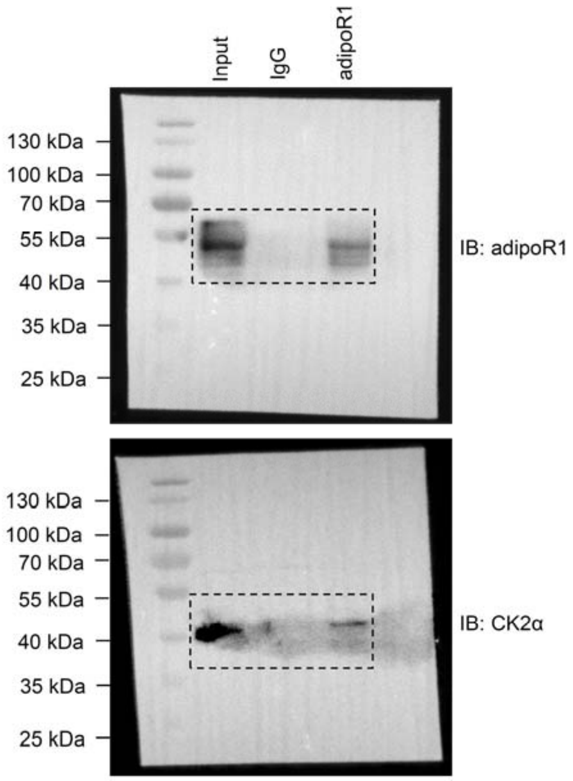


**Fig. S5: Association of CK2 alpha with the adipoR1 in mouse TGs.** Shown are the full-length pictures of the blots presented in Fig. 3E. The blots shown are representative of at least three experiments.

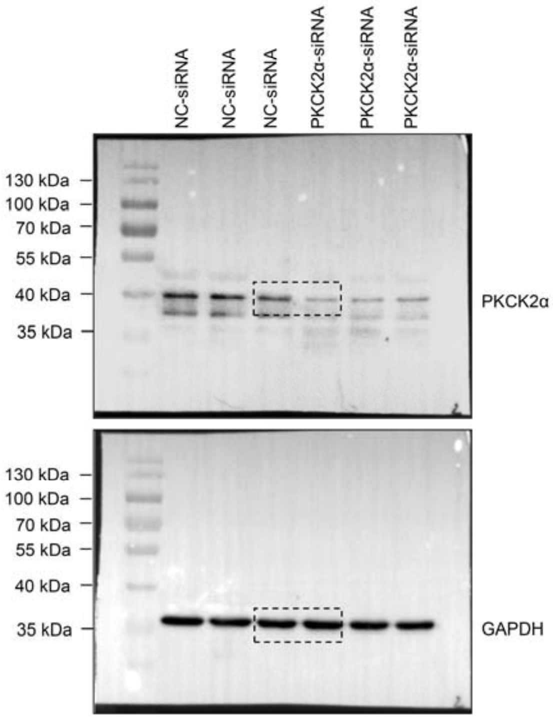


**Fig. S6: Knockdown of CK2α in mouse TGs.** Shown are the full-length pictures of the blots presented in Fig. 3I. The protein abundance of CK2α was measured using immunoblot analysis in negative control siRNA (NC-siRNA) and CK2α siRNA-treated (CK2α-siRNA) groups. The blots shown are representative of at least three experiments.

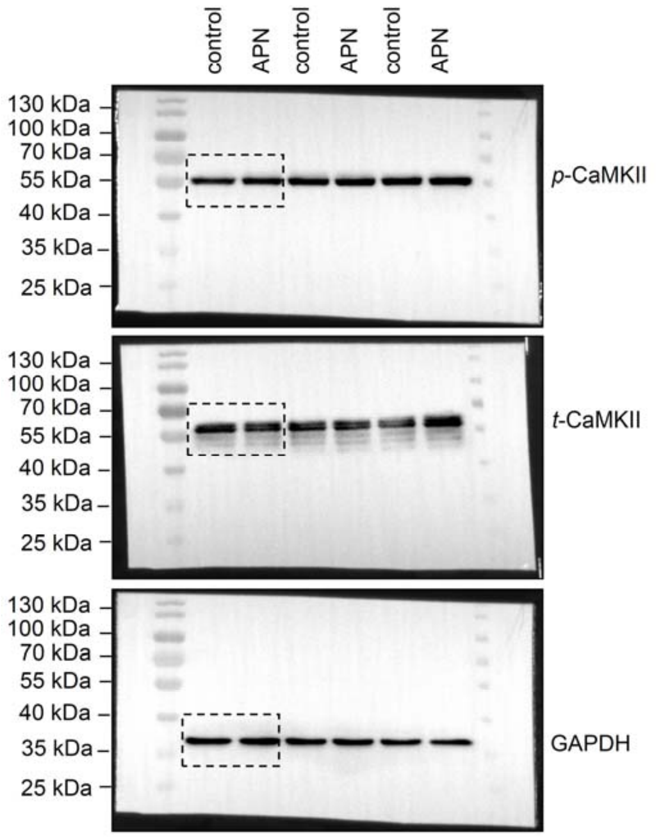


**Fig. S7: Protein expression of *p*-CaMKII and *t*-CaMKII in mouse TGs.** Shown are the full-length pictures of the blots presented in Fig. 4A. The blots shown are representative of at least three experiments.

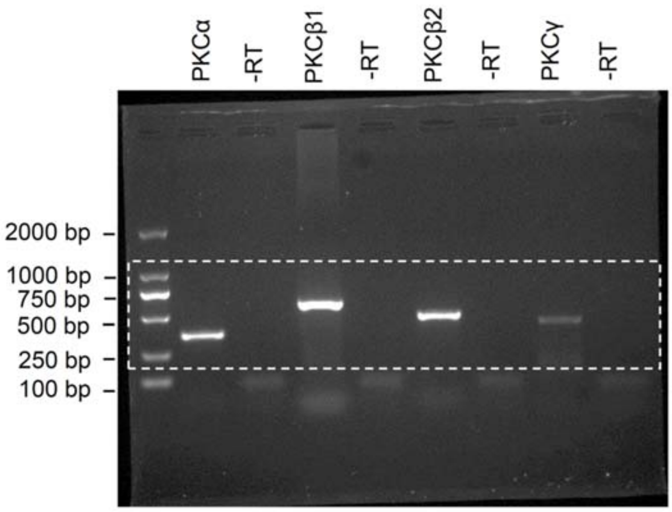


**Fig. S8: RT-PCR analysis of mRNAs of classic PKC isoforms (PKCα, PKCβ1, PKCβ2 and PKCγ) in mouse TGs.** Shown is the expanded image presented in Fig. 5A. The blot shown is representative of at least three experiments.

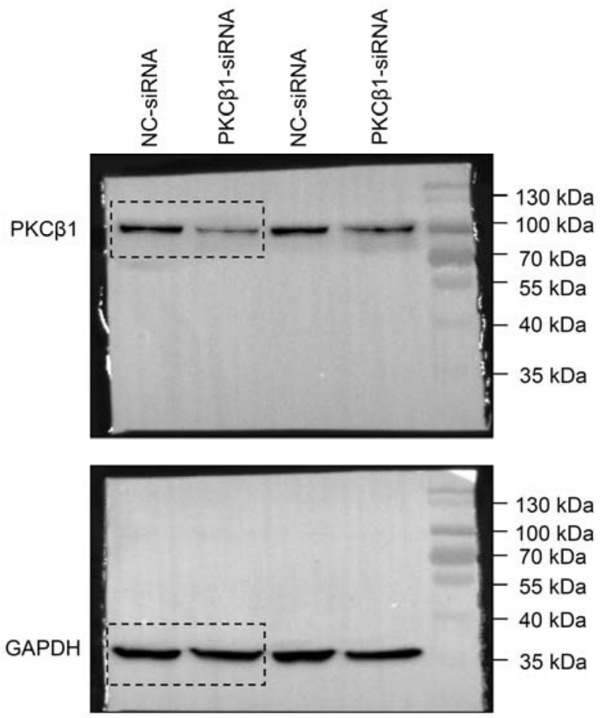


**Fig. S9: Knockdown of PKCβ1 in mouse TGs.** Shown are the full-length pictures of the blots presented in Fig. 5C. The protein abundance of PKCβ1 was measured using immunoblot analysis in negative control siRNA (NC-siRNA) and PKCβ1 siRNA-treated (PKCβ1-siRNA) groups. The blots shown are representative of at least three experiments.

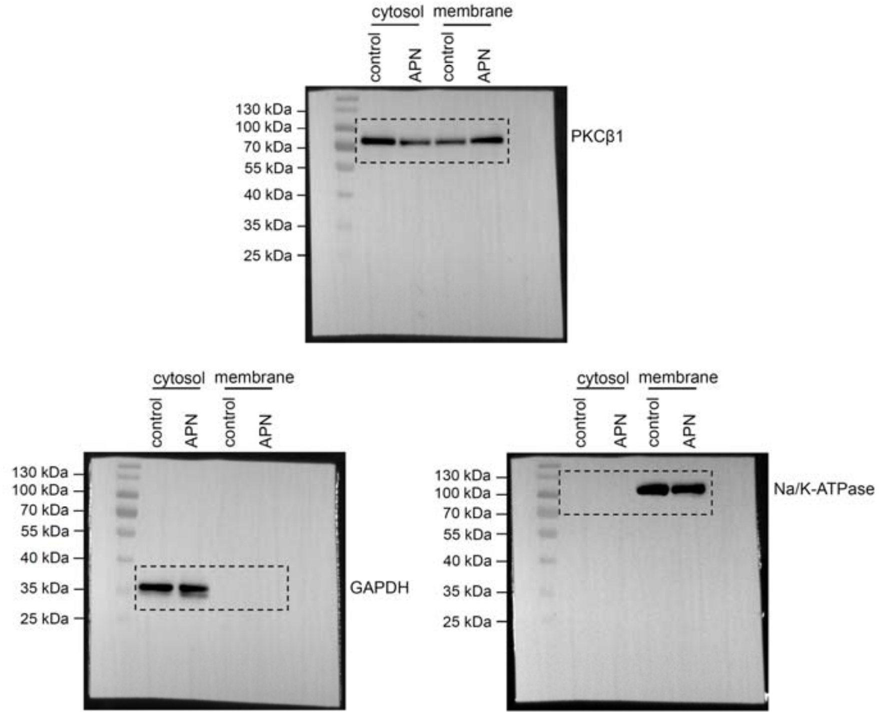


**Fig. S10: Immunoblot analysis of PKCβ1 expression in cytoplasmic and membrane fractions.** Shown are the full-length pictures of the blots presented in Fig. 5F. The blots shown are representative of at least three experiments.

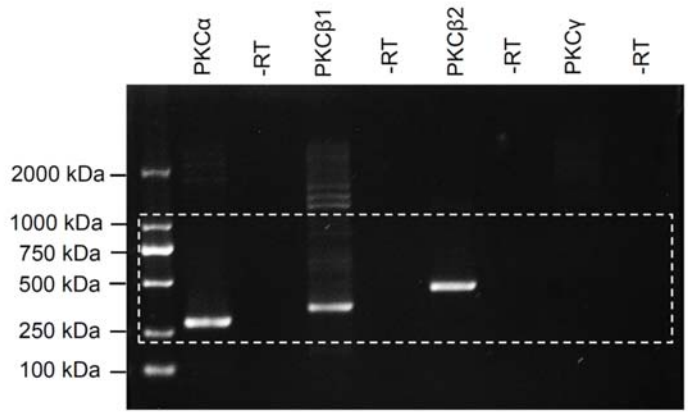


**Fig. S11: RT-PCR analysis of mRNAs of classic PKC isoforms (PKCα, PKCβ1, PKCβ2 and PKCγ) in HEK293 cells.** Shown is the expanded image presented in Fig. 6A. The blot shown is representative of at least three experiments.

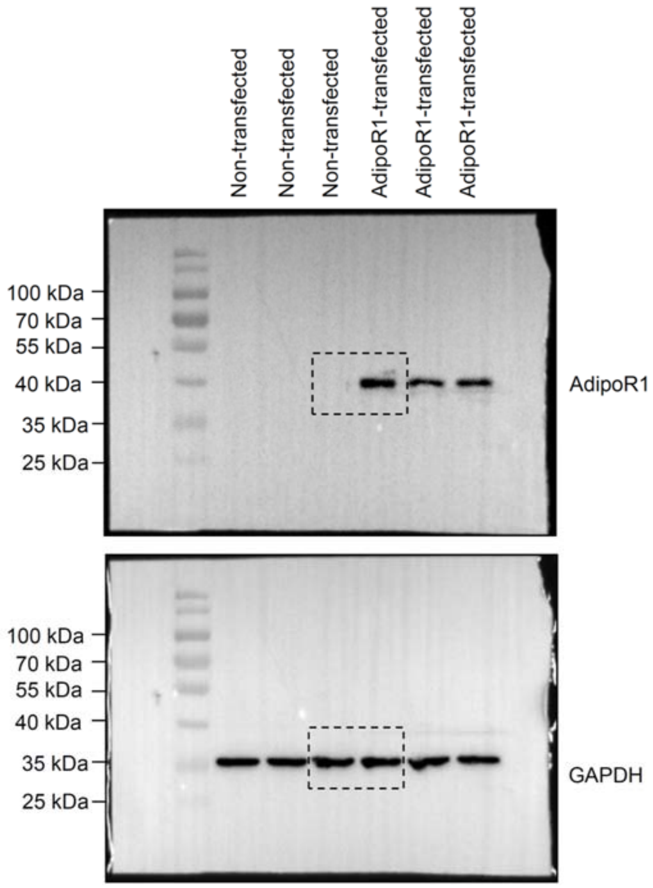


**Fig. S12: Protein expression of adipoR1 in HEK293 cells transfected with *ADIPOR1* cDNA.** Shown are the full-length pictures of the blots presented in Fig. 6B. The blots shown are representative of at least three experiments.

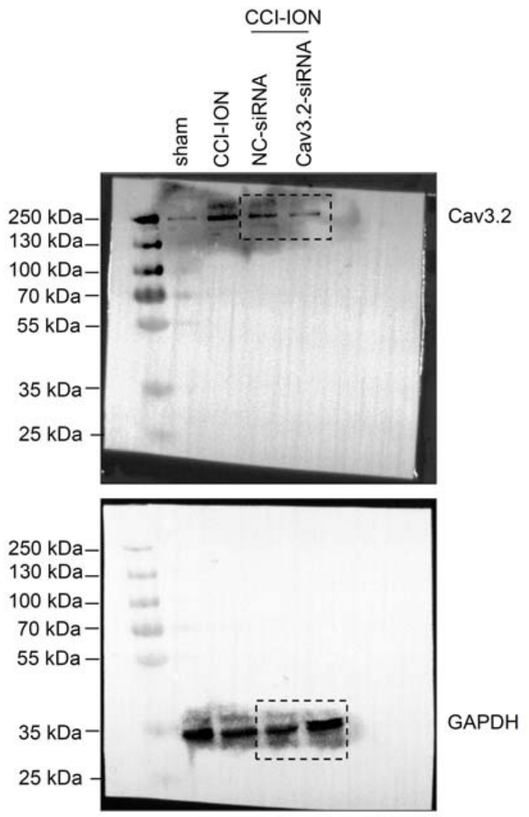


**Fig. S13: The increased expression level of Cav3.2 induced by CCI-ION was attenuated by intra-TG injection of Cav3.2-siRNA.** Shown are the full-length pictures of the blots presented in Fig. 7J. The blots shown are representative of at least three experiments.

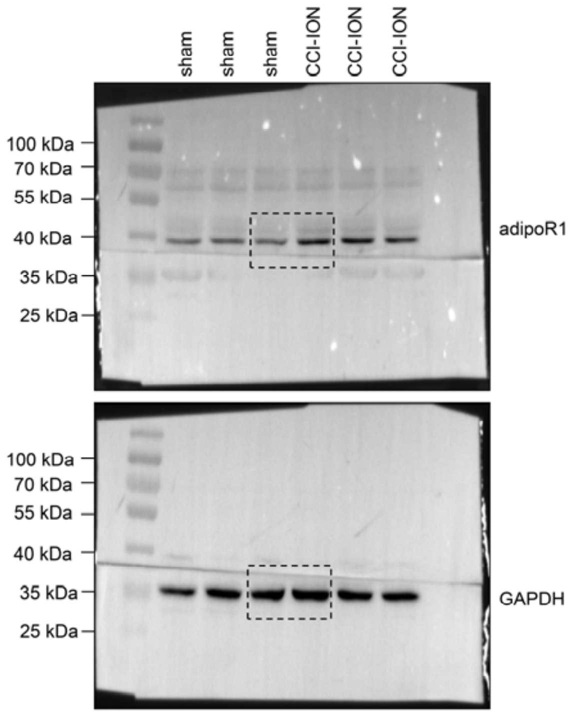


**Fig. S14: Protein expression of adipoR1 in mouse TGs after CCI-ION.** Shown are the full-length pictures of the blots presented in Fig. 8F. The blots shown are representative of at least three experiments.
